# Measuring multimorbidity beyond counting diseases: systematic review of community and population studies and guide to index choice

**DOI:** 10.1136/bmj.m160

**Published:** 2020-02-18

**Authors:** Lucy E Stirland, Laura González-Saavedra, Donncha S Mullin, Craig W Ritchie, Graciela Muniz-Terrera, Tom C Russ

**Affiliations:** 1 Edinburgh Dementia Prevention, Centre for Clinical Brain Sciences, University of Edinburgh, Kennedy Tower, Royal Edinburgh Hospital, Edinburgh EH10 5HF, UK; 2 Division of Psychiatry, Centre for Clinical Brain Sciences, University of Edinburgh, Edinburgh, UK; 3 NHS Lothian, St John’s Hospital, Howden, Livingston, UK; 4 University of Malawi College of Medicine, Blantyre, Malawi; 5 Alzheimer Scotland Dementia Research Centre, University of Edinburgh, Edinburgh, UK; 6 NHS Lothian, Royal Edinburgh Hospital, Edinburgh, UK

## Abstract

**Objectives:**

To identify and summarise existing indices for measuring multimorbidity beyond disease counts, to establish which indices include mental health comorbidities or outcomes, and to develop recommendations based on applicability, performance, and usage.

**Design:**

Systematic review.

**Data sources:**

Seven medical research databases (Medline, Web of Science Core Collection, Cochrane Library, Embase, PsycINFO, Scopus, and CINAHL Plus) from inception to October 2018 and bibliographies and citations of relevant papers. Searches were limited to English language publications.

**Eligibility criteria for study selection:**

Original articles describing a new multimorbidity index including more information than disease counts and not focusing on comorbidity associated with one specific disease. Studies were of adults based in the community or at population level.

**Results:**

Among 7128 search results, 5560 unique titles were identified. After screening against eligibility criteria the review finally included 35 papers. As index components, 25 indices used conditions (weighted or in combination with other parameters), five used diagnostic categories, four used drug use, and one used physiological measures. Predicted outcomes included mortality (18 indices), healthcare use or costs (13), hospital admission (13), and health related quality of life (7). 29 indices considered some aspect of mental health, with most including it as a comorbidity. 12 indices are recommended for use.

**Conclusions:**

35 multimorbidity indices are available, with differing components and outcomes. Researchers and clinicians should examine existing indices for suitability before creating new ones.

**Systematic review registration:**

PROSPERO CRD42017074211.

## Introduction

Multimorbidity, usually defined as the coexistence of two or more chronic conditions within an individual, is important for patient outcomes and healthcare costs. Because its prevalence is rising as populations age, multimorbidity is attracting increasing attention from the research community worldwide.^1^ More than 2800 publications on multimorbidity appeared between 1900 and 2016, 80% of which were published after 2010.^2^ As no universally agreed measure or list of diseases exists to define multimorbidity, numerous indices have been developed. These might be designed, for example, to quantify multimorbidity as a covariate in other analyses, for mortality prediction or for risk adjustment. Previous systematic reviews identified multiple indices, but no searches have been done of indices since 2009.^3^ ^4^

Multimorbidity research most often refers to a count of chronic conditions.^4^ This method does not reflect patients’ experience or the effects of different combinations or severity of diseases.^5^ Some indices, however, combine disease counts with severity measures, physiological factors, or demographic items, thereby allowing a more holistic quantification of illness burden.

The coexistence of both physical and mental illness within multimorbidity is prevalent.^6^ A 2018 report identifying priorities for multimorbidity research highlighted the need for more work into this coexistence.^1^ Researchers exploring multimorbidity will therefore increasingly need to account for mental disorders. As previous reviews of multimorbidity indices have not covered mental health in depth we identified and summarised all community based multimorbidity measures that include more than simple disease counts, paying particular attention to mental health. This review should help guide clinicians and researchers to select an appropriate index according to their requirements.

## Methods

No single accepted term describes the methods of measuring multimorbidity. In this review we use the term “index” to refer to any method of quantifying disease burden or predicting specific outcomes that includes more than a count of conditions. This could be by weighting conditions (for example, by allocating a score to each), adding other elements, or examining other variables such as drug or physiological parameters.

### Search

To capture all relevant publications we conducted a broad search. We included a variety of terms for multimorbidity derived from previous systematic reviews on this topic^3^ ^4^ and other literature discussing terminology in this area of research.^7^ We developed the search strategy iteratively with the support of an Academic Support Librarian. The final search terms are listed in the appendix (appendix eTable 1) and include multimorbidity, comorbidity, polypathology, polymorbidity, pluripathology, multi-condition, and multiple chronic conditions. The search was restricted to adults older than 18 years and to English language publications.

### Eligibility criteria

We planned to summarise reports of novel indices and were primarily interested in the original report of each index. Therefore we excluded papers that either used existing indices or measured multimorbidity using only disease counts. In the initial screening process we included only the original form of each scale and not adaptations or updates; these were found later. Records that were not original research papers, such as conference abstracts, letters, and systematic reviews, were excluded. We defined multimorbidity as multiple co-occurring conditions without reference to a specific disease, so excluded papers were those that focused on comorbidities of an index disease or on comorbidities within one disease area (such as the coexistence of several psychiatric conditions). As most people with multimorbidity are adults living in the community and managed in primary care, we excluded articles about children, animals, or people admitted to hospital or living in residential care. We included studies of hospital inpatients when the primary focus was follow-up after discharge (for example, mortality one year later). As resources were limited, we excluded papers when full texts were not available in English.

### Information sources

On 19 October 2018 we searched Medline, Web of Science Core Collection, Cochrane Library, Embase, PsycINFO, Scopus, and CINAHL Plus from inception onwards.

### Study selection

Two authors used Covidence software independently to screen titles against exclusion criteria and the subsequent abstracts against the same criteria.^8^ We then extracted the full texts of relevant abstracts for further screening. Any disagreements at the title, abstract, and full text stages were resolved by discussion, and a third author mediated unresolved conflicts. We excluded papers that referred to an existing index, but listed the indices that were used when excluding them at the abstract stage. We found the original papers describing these indices and returned them to the title screening stage. Additional relevant titles were found by reviewing previous systematic reviews on this topic, searching the bibliographies of included full text papers, and tracking their citations using Google Scholar. Emerging relevant titles were added to the screening process.

### Usage, updates, and validation

After the list of included papers had been finalised, we searched their citations on Google Scholar for updates, revisions, or adaptations as well as validation papers. When original indices were adapted and validated numerous times, we listed the original performance and principal adaptations. We did not include adaptations where the original index was translated into another language with no other changes made. To assess the popularity of each index, we took the total number of citations for each original paper from Google Scholar on 7 September 2019, as a proxy for use. We then calculated the number of citations for each whole year since publication. To retain awareness of the context of their initial design and aims, we summarised index updates separately from the original papers.

### Data collection process

We created a data extraction tool containing specific elements of interest for each original index. This tool took account of previous reviews on this topic as well as additional information relating to mental health. We used a broad definition of mental health, comprising any mental disorder, including mood disorders, dementia, delirium, and addictions as well as relevant symptoms. Many papers describe validation of the indices used so details on the size and demographic distribution of the populations under test was important.

Two authors independently extracted data from all full texts. We compared the consistency of extracted items and resolved any differences by discussion and reference to the original paper, with a third author available in case of substantially differing data extraction.

### Data items

The variables of interest during data extraction were first author, year of publication, and name of index; original purpose of the index; characteristics of the population under test, including type of data source (eg, cohort study), location, number of participants, sex and age distribution, and mean number of concurrent medical conditions (when given); components included in the index; weighting method (if any) and details of model for its development; outcome measures; information and resources required to apply the index; internal validation or comparison with another index (if applicable); external validation and performance compared with another index (if applicable); and inclusion of mental health (either in comorbidities or as outcomes).

### Risk of reporting bias in original studies

We assessed the risk of bias of study design and reporting and aimed to develop overall recommendations for index choice. The Cochrane Collaboration advises against scales that generate total numerical scores, preferring emphasis on individual papers’ performance on each criterion.^9^ After our search date the Prediction model Risk Of Bias ASsessment Tool (PROBAST) was published. It focuses on risk of bias and applicability in prediction model studies.^10^ As our search was not restricted to prediction models, it was not appropriate to apply this tool to every paper. We therefore developed our own list of criteria having referred to resources available for assessing clinical prediction indices.^11^ ^12^ ^13^ Our assessment aimed to deal with risk of selection, observer, and funding bias. The list contained 10 questions on the population tested, description of the index, statistical methods, validity, and funding. The assessment tool is available in the appendix (appendix eTable 2), including division into domains. We also included an overall impression of the papers’ risk of bias based on the Scottish Intercollegiate Guidelines Network standard, which was scored separately to the criteria rather than in an additive fashion.^14^ Two reviewers independently assessed each paper and resolved disagreements by discussion.

When choosing an index, its predictive ability and its use elsewhere are important. We generated overall recommendations taking into account the generalisability of participants, selection and clinical relevance of index components, outcome measurement, risk of reporting bias, and model evaluation. These were separated into three main categories: recommended, potentially useful (usually when indices were applicable to certain situations), and not recommended.

### Synthesis of results

We anticipated finding a wide variety of indices covering diverse outcome measures and therefore planned to summarise these narratively. Because of the range of outcomes included we did not expect to be able to perform meta-analysis. We listed performance and validity statistics as reported by the original papers or validation studies alongside each other, for comparison.

We did not expect to find indices specifically designed for measuring physical multimorbidity in relation to any aspect of mental health. Therefore, for separate narrative synthesis we planned to seek those indices that mentioned mental illnesses or symptoms, either as comorbidities or as outcomes.

### Patient and public involvement

An early draft of this paper was discussed with a lay contributor who has personal experience of multimorbidity. We incorporated her comments into the text—for example, noting in the introduction that the number of conditions a person has might not reflect their experience of health, and in the discussion her suggestions about outcomes that could be important to patients. The lay contributor also commented on a lay summary of the paper (see appendix page 43), which we amended accordingly.

## Results

### Study selection

**Fig 1:**
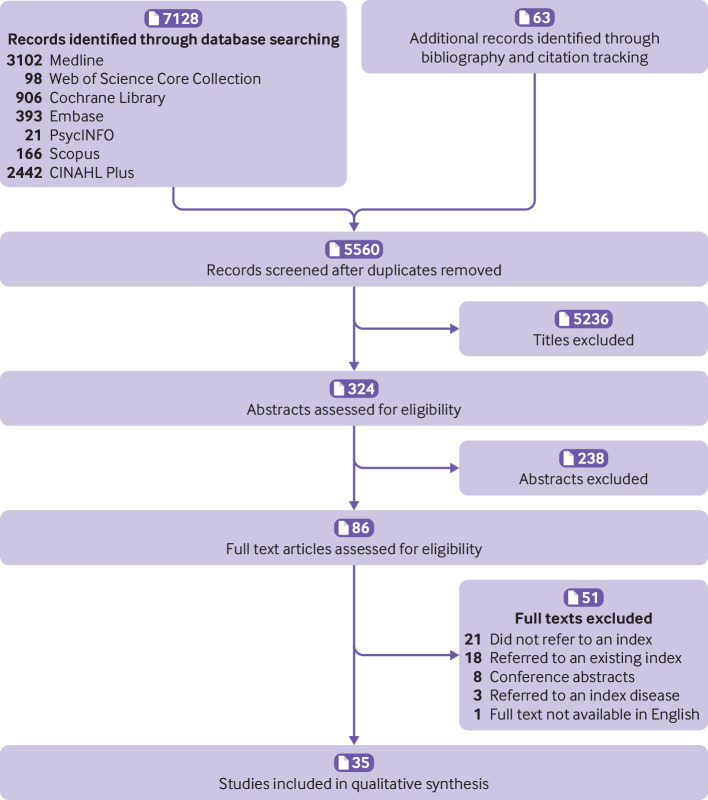
Screening process according to PRISMA

The searches yielded 7128 results. A search of bibliographies and citations identified 48 additional relevant titles, and a further 15 titles were added from the list of indices mentioned in excluded abstracts. The total number of titles was therefore 7191. Duplicates were removed using EndNote X8 and Covidence software,^8^ ^15^ leaving 5560 unique records for screening (fig 1).^16^ Overall, 5236 titles were excluded at the screening stage, leaving 324 abstracts for eligibility assessment. Of these, 86 full texts were assessed and 35 papers finally included.

### Study characteristics

Twenty articles originated from the United States^17^ ^18^ ^19^ ^20^ ^21^ ^22^ ^23^ ^24^ ^25^ ^26^ ^27^ ^28^ ^29^ ^30^ ^31^ ^32^ ^33^ ^34^ ^35^ ^36^; three from Australia^37^ ^38^ ^39^; two each from the United Kingdom,^40^ ^41^ Taiwan,^42^ ^43^ and Italy^44^ ^45^; and one each from Canada,^46^ Spain,^47^ Germany,^48^ New Zealand,^49^ Norway,^50^ and India.^51^ They were published between 1968 and 2017, with 15 (43%) published since the last systematic review on this topic in 2009.^17^ ^18^ ^19^ ^20^ ^40^ ^42^ ^43^ ^44^ ^45^ ^46^ ^47^ ^48^ ^49^ ^50^ ^51^ The mean number of participants included in the derivation populations of indices developed after 2009 was 356 906, compared with 75 491 before 2009. The newer indices primarily required access to medical records in 11 (73%) cases,^18^ ^19^ ^20^ ^40^ ^42^ ^43^ ^44^ ^45^ ^47^ ^48^ ^49^ and the remainder (4, 27%) self-report^17^ ^46^ ^50^ ^51^; 10 (50%) indices before 2009 primarily used medical records^21^ ^23^ ^26^ ^29^ ^32^ ^33^ ^34^ ^35^ ^36^ ^39^ and 10 (50%) used self-report.^22^ ^24^ ^25^ ^27^ ^28^ ^30^ ^31^ ^37^ ^38^ ^41^

The majority of papers described one final multimorbidity index, even if they tested various models in development, and four papers concluded with more than one index or measure.^23^ ^25^ ^30^ ^46^ For consistency, when articles were summarised and their quality assessed we considered each paper as a whole and noted when more than one index existed. We did not comment on models that used only unweighted disease counts, in keeping with our overall exclusion criteria.

### Index components

**Fig 2:**
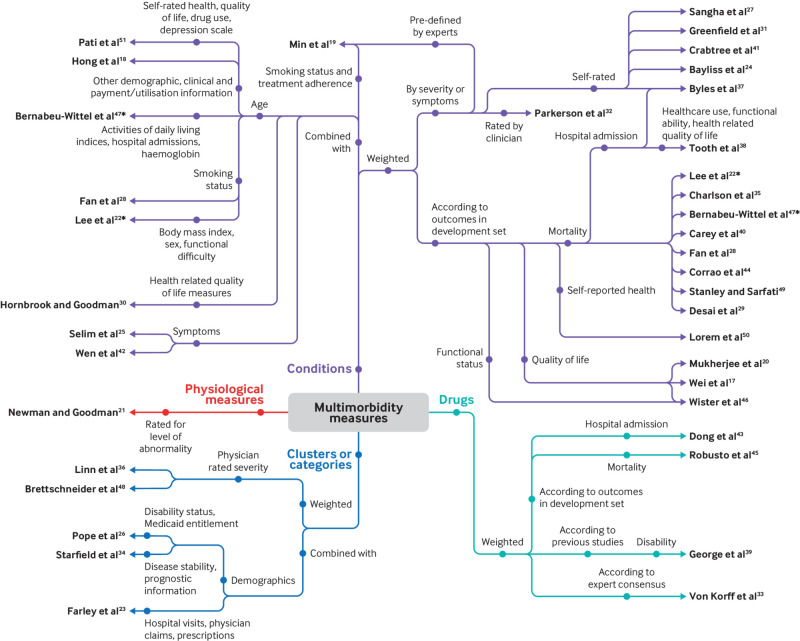
Spider diagram summarising index components. *Paper appears under more than one category

Four indices primarily used weighted drug counts to quantify multimorbidity,^33^ ^39^ ^43^ ^45^ five used diagnostic groups or clusters,^23^ ^26^ ^34^ ^36^ ^48^ and 25 included counts of diseases. Of these, 21 were weighted^17^ ^19^ ^20^ ^22^ ^24^ ^27^ ^28^ ^29^ ^31^ ^32^ ^35^ ^37^ ^38^ ^40^ ^41^ ^44^ ^46^ ^47^ ^49^ ^50^ ^51^ and nine incorporated other parameters, demographic or otherwise.^18^ ^19^ ^22^ ^25^ ^28^ ^30^ ^42^ ^47^ ^51^ Four papers used a combination of weighting and additional variables.^19^ ^22^ ^28^ ^47^ One index used physiological measurements to diagnose multimorbidity.^21^ When diagnoses were required, 14 indices took these from medical records^18^ ^19^ ^20^ ^23^ ^26^ ^29^ ^32^ ^35^ ^36^ ^40^ ^42^ ^44^ ^48^ ^49^ and 15 from self-report.^17^ ^22^ ^24^ ^25^ ^27^ ^28^ ^30^ ^31^ ^36^ ^37^ ^38^ ^41^ ^46^ ^50^ ^51^ Figure 2 is a spider diagram of the papers, displayed according to their broad category of index components.

### Outcome measures

The number of outcomes assessed in each paper ranged from none to seven. Eighteen studies measured mortality as an outcome,^19^ ^21^ ^22^ ^25^ ^28^ ^29^ ^33^ ^35^ ^36^ ^37^ ^38^ ^39^ ^40^ ^42^ ^44^ ^45^ ^47^ ^49^ 13 aimed to predict hospital admissions,^18^ ^23^ ^28^ ^31^ ^33^ ^37^ ^38^ ^39^ ^42^ ^43^ ^44^ ^45^ ^49^ 10 measured general healthcare use,^18^ ^19^ ^23^ ^25^ ^27^ ^31^ ^33^ ^34^ ^38^ ^46^ seven measured independence with daily activities or disability (with or without physical functioning),^19^ ^21^ ^24^ ^31^ ^33^ ^38^ ^41^ seven measured health related quality of life,^20^ ^25^ ^27^ ^37^ ^38^ ^46^ ^48^ five measured overall self-reported health,^24^ ^33^ ^41^ ^46^ ^50^ five measured healthcare costs,^23^ ^26^ ^27^ ^30^ ^44^ and four measured drug use.^19^ ^23^ ^27^ ^46^ Mental health was a specific outcome in three papers,^24^ ^33^ ^41^ with a further six including mental health aspects of established tools (eg, the mental component score of SF-36).^20^ ^25^ ^27^ ^37^ ^38^ ^48^ Adherence to screening programmes,^18^ specific physiological parameters,^18^ and care quality indicators^19^ were assessed by one tool each. Ten (29%) papers used cross sectional data to derive their index weighting but measured longitudinal outcomes.^19^ ^21^ ^22^ ^25^ ^33^ ^36^ ^38^ ^39^ ^42^ ^43^ Table 1 lists the papers according to their original outcomes and index components.

**Table 1:** Studies classified by components and outcomes of original versions of indices

Original outcomes	Index components					
	Conditions		Drugs (all weighted)	Categories or clusters		Physiological measures
	Weighted	With additional parameters		Weighted	With additional parameters	
**Mortality**	Corrao et al^44^	Wen et al^42^	Robusto et al^45^	Linn* et al^36^		Newman* et al^21^
	Stanley and Sarfati^49^	Min et al^19^	George et al^39^			
	Min et al^19^	Bernabeu-Wittel et al^47^	Von Korff* et al^33^			
	Carey et al^40^	Lee* et al^22^				
	Bernabeu-Wittel et al^47^	Selim et al^25^				
	Tooth et al^38^	Fan et al 8				
	Lee* et al^22^					
	Byles et al^37^					
	Fan et al^28^					
	Desai* et al^29^					
	Charlson* et al^35^					
**Healthcare use and costs**	Corrao et al^44^	Hong et al^18^	Von Korff* et al^33^	Pope* et al^26^	Farley et al^23^	
	Wister et al^46^	Min et al^19^		Starfield* et al^34^		
	Min et al^19^	Selim et al^25^				
	Tooth et al^38^	Hornbrook and Goodman^30^				
	Sangha* et al^27^					
	Greenfield* et al^31^					
**Hospital admission**	Corrao et al^44^	Wen et al^42^	Robusto et al^45^		Farley et al^23^	
	Stanley and Sarfati^49^	Hong et al^18^	Dong et al^43^			
	Byles et al^37^	Fan et al^28^	George et al^39^			
	Fan et al^28^		Von Korff* et al^33^			
	Greenfield* et al^31^					
**Independence or disability**	Min et al^19^	Min et al^19^	Von Korff* et al^33^			Newman* et al^21^
	Tooth et al^38^					
	Bayliss* et al^24^					
	Crabtree et al^41^					
	Greenfield* et al^31^					
**Self-rated overall health**	Lorem et al^50^		Von Korff* et al^33^			
	Wister et al^46^					
	Bayliss* et al^24^					
	Crabtree et al^41^					
**Health related quality of life or life satisfaction**	Wister et al^46^	Selim et al^25^		Brettschneider et al^48^		
	Mukherjee* et al^20^					
	Tooth et al^38^					
	Byles et al^37^					
	Sangha* et al^27^					
**Drug use**	Wister et al^46^	Min et al^19^			Farley et al^23^	
	Min et al^19^					
**Physical functioning**	Bayliss* et al^24^					Newman* et al^21^
	Greenfield* et al^31^					
**Mental health, depression or anxiety**	Bayliss* et al^24^		Von Korff* et al^33^			
	Crabtree et al^41^					
	Greenfield* et al^31^					
**Specific physiological measures**		Hong et al^18^				
**Quality indicators or adherence to screening**	Min et al^19^	Hong et al^18^				
		Min^19^				
**No outcomes measured**	Wei et al^17^	Pati et al^51^				
	Pati et al^51^					
	Parkerson* et al^32^					

* Studies have been subsequently updated or adapted.

### Applicability

The applicability of each index depends on study design and intended usage. Table 1 summarises index components and outcome variables. Most of the original papers (27, 77%) contained sufficient information for readers to use the index, usually with lists of included conditions with or without weighting. Of the remainder, access to additional free resources was needed in four,^30^ ^43^ ^45^ ^47^ information from proprietary scales was needed in one,^51^ and two were missing information that would allow the index to be applied.^18^ ^31^ One index was only available as proprietary software.^34^ ^52^

Some of the indices, although designed to measure multimorbidity, were developed in cohorts of people with a specific disease, and therefore this condition was not included in the list of comorbidities.^23^ ^31^ Most indices were based on diagnoses or drugs, but two indices required results of laboratory or other investigations.^21^ ^47^

The provenance of papers will affect their applicability to other settings. For example, the majority of papers came from the US where the predominant health system is commercial and healthcare costs are of interest to insurers. Some of the indices were designed with a particular population in mind, such as the questionnaire for Indian primary care. This included diseases that are less prevalent in other geographical areas, such as filariasis and tuberculosis, which might limit generalisability.^51^ Other original indices have domains that might be outdated. One example is the Charlson comorbidity index.^35^ This index assigns the maximum weight of 6 points to AIDS, but the life expectancy for HIV/AIDS in high income countries has changed considerably since the index’s publication in 1987.^53^

Although several papers mentioned outcomes as relevant to patient experience, only one clearly described involving patients in the study design, by developing their rating scale with focus groups.^37^

### Summary of evidence

Appendix eTables 3 to 6 summarise all included papers according to their index’s original purpose, components and outcome variables, and information used. The data source, location, and demographics of the population used to derive or test the measure are listed as they are relevant for context. Our overall recommendations are also included. In appendix eTable 3, the index components are weighted condition counts, in appendix eTable 4, the index components are conditions with additional parameters, in appendix eTable 5 they are weighted drug counts, and appendix eTable 6 comprises the remainder, including diagnostic groups and physiological measures.

### Weighting

The majority of indices (n=29, 83%) included some form of component weighting. Conditions, diagnostic categories, and drugs were weighted by severity or symptoms, either self-reported or defined by clinicians, or according to their associated outcomes in a derivation cohort. Different methods were used for developing each index, and disparities existed in the level of methodological detail reported. The appendix eTable 7 summarises the method for developing each model, the details provided, and baseline outcome reporting.

### Inclusion of mental health

Twenty nine (83%) of the papers contained some measure of mental health or dementia, with 18 of these including mental health markers exclusively as comorbidities (including psychotropic drugs when relevant)^17^ ^18^ ^19^ ^22^ ^26^ ^30^ ^32^ ^34^ ^35^ ^36^ ^39^ ^40^ ^42^ ^43^ ^45^ ^47^ ^49^ ^51^ and three including mental health markers as an outcome only.^24^ ^31^ ^33^ Seven measures included different aspects of mental health as both comorbidities and outcomes,^20^ ^25^ ^27^ ^37^ ^38^ ^44^ ^48^ and one paper included anxiety and depression as both a comorbidity and an outcome.^41^ In appendix eTable 8, we summarise whether each index dealt with aspects of mental health, as either comorbidities or outcomes, and how these were measured. Where papers discussed specific findings relating to multimorbidity and mental health, we present their conclusions.

### Risk of reporting bias within studies

Using our quality assessment tool, we classified six papers as high quality with little or no risk of bias in reporting^22^ ^24^ ^27^ ^32^ ^37^ ^49^ and seven as low quality with moderate to high risk of bias.^23^ ^28^ ^34^ ^35^ ^36^ ^41^ ^42^ The remaining 22 papers were of satisfactory quality. Of the five domains we assessed, the best reported were index description and funding source. Validity and statistical methods were the least well reported across all papers. Table 2 shows the scores for all papers across each domain. As we had agreed in advance to judge the overall impression without summing domain scores, the domain scores did not always lead to the same overall impression. For example, one study was given an overall impression of satisfactory with a total domain score of 6,^48^ whereas another study scored 8 and was also deemed satisfactory.^51^

**Table 2:** Overall risk of reporting bias: domain scores

Reference	Sample selection (maximum++)	Index description (maximum ++)	Statistical methods (maximum ++)	Validity (maximum +++)	Funding source (maximum ++)	Overall quality
Corrao et al^44^	-	++	+	+	++	Satisfactory
Wen et al^42^	++	++	+	-	++	Low
Stanley and Sarfati^49^	++	++	+	+	++	High
Wei et al^17^	+	++	+	+	++	Satisfactory
Robusto et al^45^	+	++	+	+	++	Satisfactory
Lorem et al^50^	+	++	++	+	++	Satisfactory
Pati et al^51^	++	++	+	++	++	Satisfactory
Hong et al^18^	++	++	+	+	++	Satisfactory
Wister et al^46^	++	+	++	-	++	Satisfactory
Brettschneider et al^48^	++	++	+	-	++	Satisfactory
Min et al^19^	-	++	+	++	+	Satisfactory
Carey et al^40^	+	++	+	+	++	Satisfactory
Dong et al^43^	+	++	+	+	++	Satisfactory
Bernabeu-Wittel et al^47^	++	++	+	+	++	Satisfactory
Mukherjee et al^20^	++	++	+	+	++	Satisfactory
Newman and Goodman^21^	++	++	+	+	++	Satisfactory
Tooth et al^38^	+	++	+	+	++	Satisfactory
George et al^39^	-	++	+	++	+	Satisfactory
Lee et al^22^	++	++	+	+	++	High
Farley et al^23^	-	-	+	+	+	Low
Byles et al^37^	++	++	+	+	-	High
Bayliss et al^24^	++	++	++	+	++	High
Selim et al^25^	+	++	+	+	++	Satisfactory
Pope et al^26^	++	++	+	+	++	Satisfactory
Sangha et al^27^	++	++	+	+++	++	High
Fan et al^28^	+	+	+	+	++	Low
Desai et al^29^	++	++	+	+	++	Satisfactory
Crabtree et al^41^	-	++	+	+	++	Low
Hornbrook and Goodman^30^	+	+	+	+	++	Satisfactory
Greenfield et al^31^	++	++	+	+	++	Satisfactory
Parkerson et al^32^	++	++	+	++	+	High
Von Korff et al^33^	-	+	+	++	++	Satisfactory
Starfield et al^34^	-	++	-	+	++	Low
Charlson et al^35^	-	++	+	+	-	Low
Linn et al^36^	-	+	+	++	-	Low

### Risk of bias across studies

It was not possible to quantify publication bias owing to the variety of methods and outcomes used. It is likely that more unpublished methods of measuring multimorbidity exist and are used in clinical practice, especially tailored to specific patient populations or available clinical information.

### Usage, performance, and validation

As a proxy for usage, we calculated the number of annual citations for each paper. The number of citations for each year since publication ranged from three^41^ to 949,^35^ with a median of 8.8 (interquartile range 5.3-16.2). This information is listed alongside measures of the indices’ performance at predicting outcomes and validation in appendix eTables 9 (indices without external validation) and 10 (externally validated indices). Sixteen original papers described designing indices within a derivation cohort and testing their ability to predict specific outcomes within a separate validation set.^20^ ^22^ ^28^ ^29^ ^30^ ^33^ ^35^ ^37^ ^38^ ^40^ ^43^ ^44^ ^45^ ^47^ ^49^ ^50^

Fourteen original papers measured an index’s performance at predicting outcomes^17^ ^22^ ^28^ ^30^ ^31^ ^32^ ^34^ ^36^ ^37^ ^38^ ^41^ ^42^ ^46^ ^48^ and 20 compared an index to an existing measure of multimorbidity.^18^ ^19^ ^20^ ^21^ ^23^ ^24^ ^25^ ^26^ ^27^ ^29^ ^33^ ^35^ ^39^ ^40^ ^43^ ^44^ ^45^ ^47^ ^49^ ^50^ Fourteen indices were validated elsewhere, of which 11 were compared with other indices^20^ ^22^ ^26^ ^27^ ^33^ ^34^ ^35^ ^36^ ^39^ ^43^ ^47^ and three only measured ability to predict outcomes.^17^ ^24^ ^32^ Among the indices that were externally validated, 11 were tested at predicting different or additional outcomes to those in the original index design.^20^ ^24^ ^26^ ^32^ ^33^ ^34^ ^35^ ^36^ ^39^ ^43^ ^47^ Some indices were tested against other indices that had been developed with different original outcomes—for example, the Charlson index where the outcome in question was admission to hospital^43^ or health related quality of life.^20^

### Updates and adaptations

Thirteen (37%) of the indices had updates or adaptations published, by either original or separate research teams. These revised versions included updated comorbidities or weights, focused on specific patient groups, or mapped a clinical index to codes for administrative data. Two of the indices are risk adjustment methods undergoing regular review and updates.^26^ ^34^ The relevant indices are listed in appendix eTable 11 alongside a summary of their adaptations and updates and any reported performance metrics. The older and widely used indices such as Charlson, Chronic Disease Score, and Cumulative Illness Rating Scale have been adapted and updated many times; we include the most cited versions. Most updated indices are broadly based on the aim and outcome measures of the original, with some exceptions.^57^ ^58^ ^59^ ^60^

Of the indices that were not updated, in some cases this was because the original index was unsuccessful at predicting specific outcomes^37^ or was not designed for use outside of the original study.^30^

## Discussion

In this review we collated descriptions of 35 distinct multimorbidity indices. The papers were diverse in study design, intended purpose, and variables included. Similarities did, however, emerge, such as index components concentrating on conditions, diagnostic categories, drug classes, or physiological measures. Mortality was the most commonly studied outcome, with healthcare use, hospital admission, functional ability, and health related quality of life as other important groups. Those that measure mortality will be of most relevance to clinicians and researchers, whereas healthcare use and costs are more useful to healthcare providers and funders, particularly in predominantly private healthcare systems. For patients, the most relevant outcomes might be quality of life or self-reported health.

### Strengths and weaknesses of this study

A major strength of this review was the use of an updated search in a rapidly expanding area of research and a focus on multimorbidity measures that specifically include mental health.

Although the medical subject heading (MeSH) term “comorbidity” has existed since 1990, a new MeSH term, “multimorbidity,” was introduced in January 2018, after our search had been designed and pre-registered.^1^ ^61^ We found that the word “indexes” was used in some titles when we had used “indices” in our search terms. One paper was found by citation tracking and had apparently been missed during our search because we had omitted the term “score.”^44^ However, we aimed to minimise the number of missed relevant papers by searching bibliographies, citations, and indices that had been mentioned in the abstracts we excluded. A limitation of this review is that we limited our search to full texts in the medical literature, excluding conference abstracts and other grey literature. This approach might have missed indices in clinical use that are either unpublished or based on guidelines from healthcare quality institutions.

In this review, we excluded papers that used simple counts of conditions to measure multimorbidity, focusing instead on indices. We chose to make this distinction because indices tend to include multiple parameters to quantify different aspects of multimorbidity and use sophisticated models to predict outcomes. However, as disease counts are the most commonly used method of measuring multimorbidity, their exclusion is a limitation of our work.^4^ The ease of applying disease counts means they are frequently used and they are comparable between studies as long as the list of candidate conditions is clear.^62^ One paper in this review reported that a count of diagnosis clusters was a better predictor of healthcare expenditure than more complex indices.^23^ Other research has drawn similar conclusions.^63^ ^64^ Reviewing the use of disease counts is outside the scope of this paper and has been discussed elsewhere.^65^ ^66^ ^67^ Simple counts of drugs have also been shown to predict healthcare use and mortality,^68^ and using these or disease counts are more practical than indices in many settings. For example, they are used in clinical care, as they do not require calculations or particular software, or in large population studies where the impact of each condition on individuals is unknown.^6^

As this review is aimed at those undertaking population or community research, we also excluded studies of people who had been admitted to hospital or live in residential care. This meant that several commonly used indices were not included in this review, such as the Elixhauser index.^69^ We did include the Charlson, PROFUND, Self-Administered Comorbidity Questionnaire, and High-Risk Diagnoses for the Elderly Scale indices as although the studies recruited hospital patients, they did so when the patients were discharged, and the main period of interest was later, in the community setting.^27^ ^29^ ^35^ ^47^

Some of the indices have been in use for many years and have several adaptations. The Charlson index is the most widely known and warranted its own systematic review within a medical specialty (critical care)^70^; we have only briefly summarised its performance and the principal adaptations. More information is available in another systematic review on this topic.^71^

Some of the indices were specifically designed for use with administrative data. These might have scored more poorly on quality assessment as our tool focused on reporting and clinical applicability. Our search also included papers that compared different measurements of multimorbidity but that were not intended for clinical use.^46^ We included these papers for completeness when they met our inclusion criteria. We aimed to find measures of multimorbidity, and our exclusion criteria included studies of comorbidity with a specific index disease. However, in two of the papers studied the indices had been developed in populations with one condition only, either hypertension^23^ or type 2 diabetes.^31^ We referred to our protocol and included these studies because their aim was to study multimorbidity rather than comorbidities of those conditions as index diseases. These papers are, however, less generalisable to the general population.

Our search was intentionally broad, using a wide range of search terms in multiple medical research databases. We included a variety of studies measuring multimorbidity from different perspectives, which is a strength over previous more specific reviews.^71^

We generated overall impressions of the risk of reporting bias and recommendations for index use, to provide a guide for researchers when choosing an index. Samples in the included studies were, however, heterogeneous, and the indices had varied purposes and components. Therefore, our recommendations are subjective. Formally comparing the predictive ability of the indices is outside the scope of this work but has been comprehensively performed by other investigators.^68^ ^72^ ^73^ ^74^

### Comparison with previous literature

The two most recent similar systematic reviews to ours did not formally assess the quality of publications.^3^ ^4^ Fifteen (43%) of the indices we included were published after 2009 and therefore would not have been found by the searches in these previous reviews. This is out of proportion to the increase in multimorbidity publications since 2010, suggesting that many recent papers have used either older measures or disease counts.^2^ A newer systematic review on this topic focused only on tools used on administrative data and conducted its search in 2012.^71^ A systematic review of multimorbidity systematic reviews, published in 2018, focused on definitions and measurement.^5^ However, it did not include a search for new indices, thereby also omitting the 15 papers published since 2010.

### Recommendations for index selection

We suggest that to select an index for clinical or research use, clinicians or researchers should first consider their desired outcomes and the information available. Box 1 summarises the process of selecting an index using this review.

Box 1 **Guide to selecting an index using this paper when designing a multimorbidity study**

**Fig 3:**
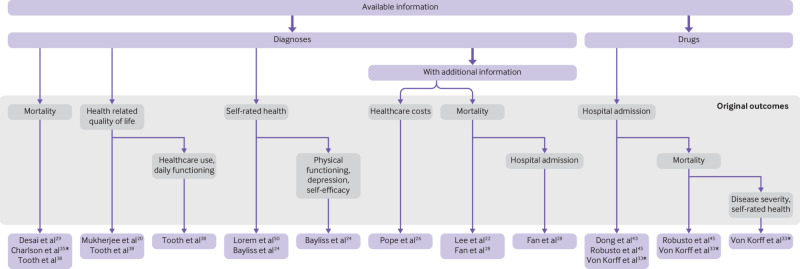
Flowchart of recommended indices organised by components and original outcomes

Identify reasons for including multimorbidity—for example, is multimorbidity important because of its association with quality of life or mortality? This will inform which outcomes of original indices are relevant
Identify the exposure variables available (eg, diagnoses, drugs)
Identify the outcomes to be measured
Use figure 3 to choose a recommended index
Use the information in the appendix (eTables 9 and 10) to compare usage and performance of any suitable indices

Appendix eTables 3-6 list our overall recommendations, divided broadly into “not recommended,” “potentially useful,” and “recommended,” and figure 3 displays the 12 indices that we recommend according to their design. The 10 indices that are not recommended could be useful for other purposes, such as recording symptoms^41^ or comparing models,^37^ but our recommendations focus on those that are practical for designing multimorbidity research.

### Conclusions

At least 35 objective measures of multimorbidity are available for people living in the community, and each of these uses different variables to generate a score or index, linked with various or no outcome measures. We found no specific index for investigating the interplay between mental and physical multimorbidity, although this issue is dealt with in a variety of ways across the measures. The array of index components and outcomes means that a validated measure exists for many applications, including clinical, research, and cost prediction. It is important when choosing an index to consider its original purpose and the outcomes for which it is validated. Given the differing methodologies of multimorbidity research, it would not be appropriate to assume that a single index could definitively measure multimorbidity in all settings. However, with this research area at risk of saturation, we propose that anyone measuring multimorbidity should study existing indices before developing new ones.

**What is already known on this topic**

It is common for people to have two or more co-occurring chronic conditions (multimorbidity)
Researchers and clinicians use many different indices to measure multimorbidity

**What this study adds**

At least 35 objective measures of multimorbidity are available for people living in the community and each of these uses different variables to generate a score or index, linked with various or no outcome measures
No specific index investigates the interplay between mental and physical multimorbidity, although this is dealt with in a variety of ways across the measures
The recommendations in this study should guide researchers to find a suitable index for their purposes

## Web extra

Extra material supplied by authors

## Notes

Ethical approval: Not required.

Data sharing: Search terms and risk of bias assessment questions are available in the appendix. Data extraction and quality assessment pro-formas are available on request from the corresponding author.

The paper’s guarantor (LES) affirms that the manuscript is an honest, accurate, and transparent account of the study being reported; that no important aspects of the study have been omitted; and that any discrepancies from the study as originally planned and registered have been explained.

Dissemination to participants and related patient and public communities: Owing to the nature of this article compiling and reviewing other researchers’ work, we have not contacted specific patient groups. We have provided a lay summary in the appendix.
