# Supplementary material for: Measuring multimorbidity beyond counting diseases: systematic review of community and population studies and guide to index choice
Source: BMJ. 2020 Feb 18;368:m160. doi: 10.1136/bmj.m160 (PMC7190061; doi:10.1136/bmj.m160)
Supplement: Supplementary file 1 — Supplementary information: Additional methods and tables [file stil050415.ww1.pdf]

eTable 1: Search strategy by database

Database	Search strategy
Web of science core collection<br><br>"Advanced search"	1. TI=(Multimorbidity or multi-morbidity or comorbidit* or co-morbidit* or polypatholog* or poly-patholog* or polymorbidit* or poly-morbidit* or multipatholog* or multi-patholog* or multicondition* or multi-condition* or pluripatholog* or pluri-patholog* or 'multiple chronic condition*' or 'morbidity burden')<br>2. TI= ((multiple or coexisting or co-existing or concurrent or comorbid or co-morbid) NEAR/2 (disease* or illness* or condition* or diagnosis or diagnoses or morbid*))<br>3. TI=(((index or indices) not ('body mass' or 'body-mass')) or (measure* or tool or instrument or categor* or rating scale* or count) or (classif* not 'international classification of disease*'))<br>4. #2 or #1<br>5. #4 AND #3<br>6. TI=(Community or outpatient* or ambulatory or ambulant or population or generalist* or 'general practi*' or 'primary care' or 'primary health*' or 'primary medic*' or 'family practi*' or 'family physician*' or 'family doctor' or 'family medic*' or 'medical practice*')<br>7. (#6 AND #5) AND LANGUAGE: (English)
Cochrane Library<br><br>"Search Manager" tab	1. ((Multimorbidity or multi-morbidity or comorbidity or polypathology or polymorbidity or poly-morbidity or multipathology or multi-pathology or multicondition or multi-condition or pluripathology or pluri-pathology or 'multiple chronic conditions' or 'morbidity burden') or ((multiple or coexisting or co-existing or concurrent or comorbid or co-morbid) NEAR/2 (disease or illness or condition or diagnosis or morbid))) NEAR/5 (((index or indices) not ('body mass' or 'body-mass')) or (measure or tool or instrument or category or rating scale or count) or (classification not 'international classification of diseases')))<br>2. (Community or outpatient or ambulatory or ambulant or population or generalist or 'general practice' or 'general practitioner' or GP or 'primary care' or 'primary health' or 'primary healthcare' or 'primary medicine' or 'primary medical' or 'family practice' or 'family practitioner' or 'family physician' or 'family doctor' or 'family medicine' or 'family medical' or 'medical practice')<br>3. #1 AND #2
Ovid MEDLINE<br><br>"Advanced Search"	1. ((Multimorbidity or multi-morbidity or comorbidit$ or co-morbidit$ or polypatholog$ or poly-patholog$ or polymorbidit$ or poly-morbidit$ or multipatholog$ or multi-patholog$ or multicondition$ or multi-condition$ or pluripatholog$ or pluri-patholog$ or 'multiple chronic condition$' or 'morbidity burden') or ((multiple or coexisting or co-existing or concurrent or comorbid or co-morbid) adj2 (disease$ or illness$ or condition$ or diagnos#s or morbid$))) adj5 (((index or indices) not ('body mass' or 'body-mass')) or (measure$ or tool or instrument or categor$ or rating scale$ or count) or (classif$ not 'international classification of disease$'))).mp.<br>2. (Community or outpatient$ or ambulatory or ambulant or population or generalist$ or 'general practi$' or GP$ or 'primary care' or 'primary health$' or 'primary medic$' or 'family practi$' or 'family physician$' or 'family doctor' or 'family medic$' or 'medical practice$').mp<br>3. 1 and 2<br>4. Animals/ not Humans/

Database	Search strategy
	5. 3 not 4<br>6. limit 5 to english language
Embase AND PsycINFO<br><br>“Advanced Search”	1. ((Multimorbidity or multi-morbidity or comorbidit$ or co-morbidit$ or polypatholog$ or poly-patholog$ or polymorbidit$ or poly-morbidit$ or multipatholog$ or multi-patholog$ or multicondition$ or multi-condition$ or pluripatholog$ or pluri-patholog$ or 'multiple chronic condition$' or 'morbidity burden') or ((multiple or coexisting or co-existing or concurrent or comorbid or co-morbid) adj2 (disease$ or illness$ or condition$ or diagnos#s or morbid$))) adj5 (((index or indices) not ('body mass' or 'body-mass')) or (measure$ or tool or instrument or categor$ or rating scale$ or count) or (classif$ not 'international classification of disease$'))).ti.<br>2. (Community or outpatient$ or ambulatory or ambulant or population or generalist$ or 'general practi$' or GP$ or 'primary care' or 'primary health$' or 'primary medic$' or 'family practi$' or 'family physician$' or 'family doctor' or 'family medic$' or 'medical practice$').mp<br>3. 1 and 2<br>4. Animals/ not Humans/<br>5. 3 not 4<br>6. limit 5 to english language
Scopus<br>“Advanced” tab	((TITLE (({multiple} OR {coexisting} OR {co-existing} OR {concurrent} OR {comorbid} OR {co-morbid}) W/1 (disease* OR illness* OR condition* OR diagnos?s OR morbidit*))) OR (TITLE ({multimorbidity} OR {multi-morbidity} OR comorbidit* OR co-morbidit* OR polypatholog* OR poly-patholog* OR polymorbidit* OR poly-morbidit* OR multipatholog* OR multi-patholog* OR multicondition* OR multi-condition* OR pluripatholog* OR pluri-patholog* OR {multiple chronic condition*} OR {morbidity burden}))) AND (TITLE ((measure* OR "tool" OR "instrument" OR category* OR rating AND scale*) OR (classif* AND NOT {international classification of disease*}) OR (({index} OR {indices}) AND NOT ({body mass index} OR {body-mass index})))) AND (TITLE-ABS-KEY ({community} OR outpatient* OR {ambulatory} OR {ambulant} OR {population} OR generalist* OR {general practi*} OR gp* OR {primary care} OR {primary health*} OR {primary medic*} OR {family practi*} OR {family physician*} OR {family doctor} OR {family medic*} OR {medical practice*})) AND (LIMIT-TO (LANGUAGE , "English "))
CINAHL Plus	(TX (((Multimorbidity or multi-morbidity or comorbidit* or co-morbidit* or polypatholog* or poly-patholog* or polymorbidit* or poly-morbidit* or multipatholog* or multi-patholog* or multicondition* or multi-condition* or pluripatholog* or pluri-patholog* or 'multiple chronic condition*' or 'morbidity burden') or ((multiple or coexisting or co-existing or concurrent or comorbid or co-morbid) N2 (disease* or illness* or condition* or diagnos#s or morbid*))) N5 (((index or indices) not ('body mass' or 'body-mass')) or (measure* or tool or instrument or category* or rating scale* or count) or (classif* not 'international classification of disease*')))) AND TX((Community or outpatient* or ambulatory or ambulant or population or generalist* or 'general practi*' or GP* or 'primary care' or 'primary health*' or 'primary medic*' or 'family practi*' or 'family physician*' or 'family doctor' or 'family medic*' or 'medical practice*')))<br>Limiters: Age group: all adult; Language: English

eTable 2: Risk of bias assessment tool questions, by domain

Questions 1-9 marked as follows: Yes + No or not applicable -

Participant selection (maximum ++)

1. Are the patient/population demographics of this study clearly described?
2. Are the patient/population demographics representative (eg. Including an appropriate proportion of genders, socioeconomic status etc)

Index description (maximum ++)

3. Are the variables included in the index clearly defined?
4. If the index uses a list of diseases, does it describe the selection process for this list?

Statistical methods (maximum ++)

5. Are the statistical methods used clearly described?
6. Is a sample size calculation included?

Validity (maximum +++)

7. When outcomes were included, were outcome raters blinded to the variables used in the index?
8. Was there a test for inter-rater or test-retest reliability of the index?
9. Was the index validated, either in this paper or elsewhere?

Funding source (maximum ++)

10. Is there a statement of funding or conflicts of interest?

Yes: no likely conflict ++

Yes: possible conflict +

No statement -

Overall quality criteria (based on SIGN Guidelines)[1]

High: Majority of criteria met. Little or no risk of bias

Satisfactory: Most criteria met. Some flaws in the study with an associated risk of bias

Low: Either most criteria not met, or significant flaws relating to key aspects of study design.

eTable 3: Data extracted from original papers where weighted conditions are the index components, displayed in chronological order, with overall recommendation for use

Publication and name of index	Original purpose	Data source and location	Population demographics	Index components	Information used	Original outcomes measured	Recommendation
**Corrao 2017 [44] Multisource Comorbidity Score**	Predicting mortality, hospitalisation and healthcare costs	Population-based retrospective cohort study of people aged ⩾ 50 years from administrative databases. Italy	Derivation: n=500 000 Validation sets: n=4×500 000 All aged ⩾50 years; no further details	34 conditions taken from diagnosis codes and medication use, weighted by association with mortality in derivation set	Inpatient medical records and outpatient prescriptions. Weights and list of conditions given in paper	One- and five-year mortality and hospital admissions, two-year hospital costs	Potentially useful for predicting mortality; requires further evaluation
**Stanley 2017 [49] Measuring multimorbidity (M3) Index**	Predicting mortality	Routinely collected public healthcare data. New Zealand	Derivation: n=2 331 645, 52.2% women Validation: n=1 000 166, 52.2% women	55 conditions, weighted by association with mortality in derivation set	Routinely collected hospitalisation data. Weights and list of conditions given in paper	One-year mortality and one-year non-maternity hospital admission	Potentially useful for predicting mortality; requires further evaluation
**Wei 2016 [17] Multimorbidity Weighted Index**	Measuring disease burden	Prospective cohort studies of nurses and health professionals. USA	n=216 890, mean age 55 years, 80.1% women, mean 3.3 chronic conditions	74 self-reported conditions, weighted by physical functioning domain of SF-36	Self-reported diagnoses. Weights and list of conditions given in paper	None tested in this paper	Potentially useful for predicting physical function; requires further evaluation
**Lorem 2016 [50] Health Impact Index**	Estimating levels of self-reported health	Longitudinal cohort study of all adults aged over 25 years within one region. Norway	Derivation: n=26 684, 52.6% women. Validation: n=804, 55% women. Age distribution not given	19 conditions, weighted according to association with self-reported health in derivation set	Self-reported diagnoses. Weights and list of conditions given in paper	Self-reported health, asked in survey question with four-point self-rating scale	Recommended for measuring disease burden. Needs external validation
**Wister 2015 [46]**	Predicting life satisfaction, perceived health,	Subsample of cross-sectional study of adults	n=16 369, 54.9% women, mean 2.8 conditions	19 self-reported chronic conditions. Six models	Self-reported diagnoses from survey. Weights and list	Score on Satisfaction with Life Scale, self-reported health	Not recommended. Primarily comparison of methods; not designed for external use

Publication and name of index	Original purpose	Data source and location	Population demographics	Index components	Information used	Original outcomes measured	Recommendation
	healthcare and medication use	aged over 65 years. Canada		examined: three count-based, others weighted according to Health Utility Index with and without age and gender, OARS ADL scale	of conditions given in paper	(one question), self-reported health professional visits, self-reported daily medication use	
**Carey 2013 [40] QOF Comorbidity Score**	Mortality prediction	Retrospective cohort study of anonymised primary care records of patients aged ⩾60 years from 375 GP practices. UK	Derivation: n=317 876, mean age 71.6 years, 51.4% women. Validation: n=335 904, mean age 71.6 years, 51.4% women	15 chronic conditions with subgroups for some, weighted based on mortality in derivation set	Primary care records. List of conditions and weighting given in paper	One-year mortality	Potentially useful for predicting mortality in primary care. Needs external evaluation
**Mukherjee 2011 [20] Health-Related Quality of Life Comorbidity Index (HRQL-CI) ***	Predicting health-related quality of life (HRQL)	Medical records from participants in population survey. USA	Derivation: n=12 713, 61.6% women. Validation: n=12 812, 60.5% women. No other details given	26 Clinical Classification Codes, weighted by association with outcomes in derivation set and by clinical judgement	Diagnoses from primary and secondary care health records. Lists of conditions and weighting in original paper	SF-12 health outcome survey, two single-item self-report health status questions	Recommended for predicting health-related quality of life
**Tooth 2008 [38]**	Prediction of mortality, health service use, ADL independence and HRQL	Longitudinal population-based survey of women. Australia	Derivation: 5,217, mean age 74.9 years, 100% women, median 2 chronic conditions. Validation: 5,217, mean age 74.9 years, 100%	17 self-reported conditions, of which two include severity scale	Self-reported diagnoses. Conditions and weights listed in original paper	Six-year mortality and self-reported measures from survey follow-up: annual healthcare use, assistance with ADL, SF-36	Recommended for predicting less well studied outcomes. Needs further evaluation in sample including men

Publication and name of index	Original purpose	Data source and location	Population demographics	Index components	Information used	Original outcomes measured	Recommendation
			women, median 2 chronic conditions				
**Byles 2005 [37]**	Prediction of quality of life, mortality and hospitalisation	Veterans and war widows aged ⩾70 years enrolled in RCT. Australia	Derivation: n=869, median age 76 years, 45% women. Validation: n=434, median age 76, 47% women. Mean 7 conditions in both cohorts	25 conditions, each with self-reported severity rating. Weighted depending on outcomes in derivation cohort	Self-reported diagnoses and severity from survey. List of conditions and weighting given in this paper	Two-year mortality, hospital admission, quality of life measured using SF-36	Not recommended; as authors note, using one model is not effective at predicting multiple outcomes
**Bayliss 2005 [24] Disease Burden Morbidity Assessment ***	Quality of life prediction	Postal survey sent to stratified random sample of one healthcare provider's members aged over 65 years. USA	n=156, mean age 75 years, 49.4% women, mean 5.9 chronic conditions	25 chronic conditions (self-report), each weighted by self-reported interference with daily activities	Self-reported diagnoses and severity from survey. List of conditions in original paper	Overall health status, physical functioning (both from SF-36), depression, self-efficacy	Recommended for predicting self-rated health, depression, physical functioning and self-efficacy
**Sangha 2003 [27] Self-Administered Comorbidity Questionnaire ***	Predicting resource utilisation and health status	Randomly selected patients aged ⩾50 years admitted to general medical and general surgical inpatient units. USA	n=170, mean age 65.3 years, 55% women	13 conditions and space for ⩽ 3 free-text entries, weighted by patient-reported impact on daily life	Self-reported diagnoses and severity weighting in questionnaire. All domains included in paper	Resource utilisation during hospital stay; at one-year follow up: SF-36 and patient-reported visits to physicians and medication use	Potentially useful for predicting HRQL and healthcare costs through self-report methods in research setting
**Desai 2002 [29] High-Risk Diagnoses for**	Predicting mortality	Two prospective cohort studies	Derivation:	10 conditions, weighted by relative risk for	Diagnoses from inpatient discharge	One-year mortality	Recommended for predicting mortality – most relevant in

Publication and name of index	Original purpose	Data source and location	Population demographics	Index components	Information used	Original outcomes measured	Recommendation
**the Elderly Scale * (HRDES)**		within hospital general medicine service. USA	n=524, mean age 78.7 years, 56% women Validation: n=852, mean age 79.7 years, 61% women	mortality in derivation set	records. Lists of conditions and weighting in original paper		inpatients as sepsis is included
**Crabtree 2000 [41] Comorbidity Symptom Scale (CmSS)**	Classification of comorbidities for longitudinal study	Outpatient cataract and geriatric day hospital patients. UK	Derivation: n=50, all aged ⩾65 years (no other details given) Validation: n=183, median age 78.0 years, 68.3% women, mean 6 conditions	22 conditions, some with details about symptoms, self-report severity scale	Self-reported diagnoses, symptoms and severity in questionnaire. Item list included in paper	Activities of Daily Living (NEADL), perceived health status (GHQ-28), anxiety and depression (HAD)	Potentially useful for gathering information on symptoms but not recommended as index due to small sample and minimal validation
**Greenfield 1995 [31] Total Illness Burden Index (TIBI)***	Measuring functional status and quality of life	Participants of longitudinal cohort study with type II diabetes. USA	n=1,738, mean age 66.3, 50.8% women	15 conditions weighted for symptom severity	Self-reported conditions in questionnaire; list of conditions but not symptom severity given in original paper	Physical function, role function according to SF-36, mental health index. Disability days, doctor visits and hospitalisations within six months	Not recommended. Participants all had diabetes; list of weights not readily available
**Parkerson 1993 [32] Duke Severity of Illness Checklist (DUSOI)***	Controlling for overall illness severity in research	Medical records of convenience sample of primary care attendees aged 18-65 years. USA	n=414, mean age 40.5, 58.7% women, mean 1.9 chronic conditions	All conditions noted in medical records, weighted by rater-judged symptoms, complications,	Diagnoses, symptoms, complications, prognosis and treatability from primary care medical records.	None	Potentially useful for establishing medical history in clinical setting but has no limits on what constitutes conditions so limited applicability in research

Publication and name of index	Original purpose	Data source and location	Population demographics	Index components	Information used	Original outcomes measured	Recommendation
				prognosis and treatability	Sample checklist in original paper		
**Charlson 1987 [35] Charlson Index***	Classification of comorbidities for longitudinal studies	Derivation: All patients admitted to one hospital during one month, hospital records data collected at discharge. Validation: all women receiving treatment for primary breast cancer at a single hospital. USA	Derivation: n=604, mean 1.68 conditions. Validation: n=685, 100% women. No other details given	19 conditions, weighted according to mortality in derivation set	Diagnoses from inpatient discharge records. Included conditions and weighting listed in original paper	One-year mortality	Recommended for predicting mortality due to widespread use despite possible flaws in methods. Original weights are outdated; recommend using Quan update [54] (see eTable 11)

* Indices that have an updated or modified version available

eTable 4: Data extracted from original papers where index components include conditions with additional information

Publication and name of index	Original purpose	Data source and location	Population demographics	Index components	Information used	Original outcomes measured	Recommendation
**Wen 2017 [42] Multimorbidity Frailty Index**	Predicting mortality and hospital use	Retrospective cohort study of people aged 65-100 years using national health records database. Taiwan	n=86 133, mean age 73.4 years, 50.2% women	32 codes from ICD-9-CM, of which three are symptoms and 29 diagnoses	Inpatient and outpatient claims records. List of conditions given in paper	Mortality, unplanned hospitalisations and ICU admissions at one, five and eight years	Not recommended: not evaluated and is not a frailty index as it claims
**Pati 2016 [51] Multimorbidity Assessment Questionnaire for Primary Care (MAQ-PC)**	Quantifying multimorbidity for epidemiological work	Systematic random sample of adult primary care attendees. India	n=103, mean age 45.0 years, 45% women, mean 1.6 chronic conditions	18 self-reported conditions, three open-ended diagnosis questions all with self-reported severity, medication use, self-reported HRQL, SF-12, PHQ-9, healthcare utilisation, demographic variables	Self-reported diagnoses and medication from questionnaire. Healthcare use and demographics from medical records. PHQ-9 (free), SF-12 (paid licence)	None reported	Potentially useful for information gathering. Conditions relevant to low-income setting
**Hong 2015 [18] estimated Physician Defined Complexity (ePDC)**	Risk stratification for resource allocation	Electronic health data from adults in primary care research network. USA	n=143 372, mean age 49 years, 57.4% women (split into two-thirds for derivation and one-third for validation)	24 variables including 9 chronic conditions, HbA1c, demographic, healthcare utilisation and medication information	Electronic primary care health records: demographics, diagnoses, HbA1c, appointment and billing data, algorithm to define diabetes (information not	Hospitalisation, emergency visits, adherence to cancer screening programmes, HbA1c, LDL cholesterol in patients with	Not recommended: not usable as information available is incomplete

Publication and name of index	Original purpose	Data source and location	Population demographics	Index components	Information used	Original outcomes measured	Recommendation
					provided; tried to contact author without success)	cardiovascular disease	
**Min 2013 [19] Geriatric CompleXity of Care Index (GXI)**	Predicting complicated ambulatory care including polypharmacy	Medical records from participants aged ⩾75 years in cohort study. California, USA	n=644, mean age 81.2, 66.4% women, mean 3.6 chronic conditions	25 conditions and behavioural states, weighted by expert opinion on contribution to complexity	Diagnoses from primary care medical records. List of conditions and weights in original publication	Five-year mortality and functional decline, two provider visit variables, polypharmacy, and number of eligible quality indicators	Potentially useful in older people but needs external evaluation in larger sample
**Bernabeu-Wittel 2011 [47] PROFUND**	Mortality prediction	Consecutive patients with ⩾2 conditions aged over 18 years from internal and geriatric medicine in 33 hospitals, recruited for cohort study at discharge or in community. Spain	Derivation: n=757, mean age 79 years, 45.7% women. Validation: n=768, mean age 78.8 years, 45.3% women	Age, four conditions, haemoglobin, Barthel Index (ADLs), care giver status, hospitalisations in last year. Weighted depending on mortality in derivation cohort	Number of hospitalisations and diagnoses from secondary care records or self-report, age, care giver status. Barthel Index (available for free). Functional status according to NYHA/MRC (available free), haemoglobin. Weighting in this paper.	One-year mortality	Potentially useful for predicting mortality but requires specific components that may not be commonly available
**Lee 2006 [22] ***	Mortality prediction	One wave of cohort study of adults aged over 50 years. USA	Derivation: n=11 701, mean age 67 years, 57% women. Validation: n=8,009, mean	Age, sex, four chronic conditions, smoking, BMI, four functional measures,	Self-report in survey: demographics, diagnoses, smoking status. BMI. Questions	Four-year mortality	Recommended for predicting mortality as long as functional measures are available

Publication and name of index	Original purpose	Data source and location	Population demographics	Index components	Information used	Original outcomes measured	Recommendation
			age 67, 56% women	weighted based on mortality	on functional ability and weights both listed in paper		
**Selim 2004 [25]**	To assess HRQL and predict healthcare utilisation and mortality	Postal survey sent to participants in longitudinal veterans' cohort study. USA	n=2,425, mean age 64, 0% women, mean 6.3 chronic conditions	Combination of 36 self-reported conditions and symptom list, separated into physical and mental conditions. (Three other models purely count-based)	Self-reported diagnoses and symptoms (survey interview). Conditions and symptoms listed in paper	SF-36, number of outpatient visits, 35-week mortality	Not recommended: not designed as index, includes several models and some methods are unclear
**Fan 2002 [28] Seattle Index of Co-morbidity**	Predicting mortality and hospitalisation	Participants of prospective cohort study aged ⩾50 years, various outpatient centres, USA	Derivation: n=5,469, mean age 67.8 years, 2.6% women, mean 3.8 chronic conditions. Validation: n=5,478, mean age 67.8, 2.7% women, mean 3.8 chronic conditions	7 chronic conditions, weighted based on mortality in derivation set, age, smoking status	Self-reported diagnoses and smoking status (from survey). Age from records. List of conditions and weighting given in paper	All-cause mortality, hospitalisation	Recommended for predicting mortality where medical history and smoking status available
**Hornbrook 1996 [30]**	Prediction of healthcare expenditure	Postal survey sent to two random samples of patients from one primary healthcare provider. USA	n=7,739, mean age 42.2 years, 55.4% women (Split in half at random to test different models)	Five models including various combinations of demographics, RAND-36 scales and self-report of six chronic diseases	Self-reported diagnoses, demographics from administrative records, RAND-36 (available free online)	One year's total health plan expenditure	Not recommended; as authors state it is exploratory work and do not identify one model for external use. Useful for comparing cost prediction models

* Indices that have an updated or modified version available

eTable 5: Data extracted from original papers where index components are weighted drug counts

Publication and name of index	Original purpose	Data source and location	Population demographics	Index components	Information used	Original outcomes measured	Recommendation
**Robusto 2016 [45] Drug-Derived Complexity Index**	Predicting mortality and hospitalisation	Population-based retrospective cohort study using record linkage. Italy	Derivation: n=999 391, mean age 60.2 years, 53.7% women. Validation: n=999 557, mean age 60.2 years, 53.7% women	19 classes of drug, weighted based on mortality in derivation set	Community prescribing records organised by WHO ATC codes (available for free). Weights and lists of drugs given in paper	One-year and overall mortality, unplanned hospitalisation	Recommended for measuring multimorbidity using medication data to predict mortality. Needs external validation
**Dong 2013 [43] Pharmacy-Based Disease Indicator**	Prediction of hospitalisation	Routinely collected pharmacy data from adults aged ⩾18 years from longitudinal health insurance database. Taiwan	Derivation: n=697 823, mean age 43.4, 51.4% women. Validation: n=714 072, mean age 43.6, 51.6% women	37 drug categories, weighted based on hospitalisation in derivation cohort	Prescribing records from primary and secondary care, organised by WHO ATC codes. Weights and lists of drugs in original paper	One-year hospitalisation	Recommended for predicting hospitalisation using prescribing data
**George 2006 [39]**	Prediction of mortality and hospitalisation	Participants of RCT taking ⩾ 3 medications	n=317, mean age 71.8 years, (gender	Drugs corresponding to 20 conditions,	Prescribing records from inpatient	Hospital readmission or	Potentially useful but requires validation in larger studies

Publication and name of index	Original purpose	Data source and location	Population demographics	Index components	Information used	Original outcomes measured	Recommendation
**Medication-based Disease Burden Index (MDBI)**		(and with other risk factors) discharged from two tertiary care hospitals. Australia	breakdown not given), mean 10.4 medications	weighted based on burden of disease studies	discharges. List of drugs and weights given in this paper	death within 12 weeks	
**Von Korff 1992 [33] Chronic Disease Score***	Measuring chronic disease using pharmacy data	Routinely collected prescription data from various healthcare providers. USA	Derivation samples: n=219, n=722, n=1,016, n=2,247. Validation: n=122 911. Overall demographics not given	25 classes of drug, weighted according to expert consensus	Drugs from medical or pharmacy records. List of drugs and weighting given in original paper	Physician rating of physical disease severity (pilot sample). One-year mortality and hospitalisations. In smaller samples, measured self-rated health, chronic pain, functional disability, depression, anxiety and somatisation according to SCL-90-R	Recommended for measuring disease severity and predicting mortality and hospitalisations using drug data. Advise using updated version Rx-Risk for newer list of drugs [55] (see eTable 11)

* Indices that have an updated or modified version available

eTable 6: Data extracted from original papers where index components are diagnostic groups or physiological measures

Publication and name of index	Original purpose	Data source and location	Population demographics	Index components	Information used	Original outcomes measured	Recommendation
**Brettschneider 2013 [48]**	Quality of life prediction	Cohort study of randomly selected general practice patients. Subset studied had ⩾3 conditions. Germany	n=3,189, mean age 74.4 years, 59.3% women, mean 7 chronic conditions	42 diagnostic groups, each with severity rating	Primary care diagnoses and severity rating of each condition according to participant's GP. List of conditions given in paper	Health-related quality of life according to EQ-5D	Potentially useful for assigning condition weights in other studies
**Newman 2008 [21] Physiologic Index of Comorbidity ***	Predicting mortality and disability	Measurements taken as part of longitudinal cohort study of people aged ⩾ 65 years. USA	n=2,928, mean age 74.5, 57.8% women	Five non-invasive physiological parameters, all graded for abnormality on three-point scale	Carotid ultrasound, pulmonary function testing, brain magnetic resonance scan, serum cystatin-C, and fasting glucose. From cohort study but could be taken from medical records. Weighting given in this paper	Nine-year mortality, mobility disability, ADL disability	Not recommended: requires very specific components. Updated version Healthy Ageing Index more practical [56] (see eTable 11)
**Farley 2006 [23]**	Prediction of healthcare expenditure	Electronic health records of adults in managed care organisation aged ⩾18 years who filled a prescription for an anti-hypertensive. USA	n=20 378, mean age 49.0 years, 47% women, mean 7.1 diagnosis clusters	Two separate final models: Count of hospital visits, physician claims and unique prescriptions with and without counts of 119 ICD-9-CM diagnostic clusters. With and	Data on physician and hospital claims, diagnoses, prescriptions and demographics from health records	One-year healthcare expenditure	Not recommended for external use, but provides evidence comparing models with disease counts

Publication and name of index	Original purpose	Data source and location	Population demographics	Index components	Information used	Original outcomes measured	Recommendation
				without age and gender			
**Pope 2004 [26] The Centers for Medicare and Medicaid Services' Hierarchical Condition Category (CMS-HCC) ***	Predicting medical expense risk	Fee-for-service claims of 5% sample of population covered by large healthcare provider (with specific subsample of nursing home residents). USA	n=1 337 887, no further details given	70 hierarchical condition categories, gender, age, Medicaid enrolment, disability status, interactions between diseases and between diseases and disability status	Diagnosis categories and demographic information from primary and secondary healthcare records. Conditions listed in paper	Healthcare expenditure	Recommended for predicting healthcare expenditure in USA setting
**Starfield 1991 [34] Ambulatory Care Groups***	Predicting healthcare utilisation and costs	Routinely collected data from outpatients covered by five healthcare providers. USA	Total n=106 551 including adults and children. No further details given	34 diagnostic groups, weighted according to recorded stability and collapsed into 12 overall groups	Requires access to proprietary software. Diagnoses and severity from primary care medical records, demographics from administrative or medical records. Diagnostic groups listed in original paper	Annual number of healthcare visits and healthcare charges	Potentially useful for predicting costs in USA setting but needs proprietary software

Publication and name of index	Original purpose	Data source and location	Population demographics	Index components	Information used	Original outcomes measured	Recommendation
**Linn 1968 [36] Cumulative Illness Rating Scale (CIRS) ***	Assessing physical impairment for research	Study of adults aged over 55 years. USA	n=472, no further details given	13 disease areas, each scored for severity by assessing physician	Diagnoses and severity from physician interview or medical records. Body systems listed in original paper	Not assessed in detail but briefly mentions correlation with deaths, vital organ involvement and number of previous illnesses	Potentially useful for information gathering in clinical and research settings although somewhat subjective

* Indices that have an updated or modified version available

Abbreviations in eTables 3 to 6:

ADL: Activities of Daily Living
APR-DRGs: All Patient Refined Diagnosis Related Groups
BMI: body mass index
EQ-5D: EuroQol five-dimension measure of health status
GHQ-28: 28-Item General Health Questionnaire
GP: General Practitioner
HAD: Hospital Anxiety and Depression Scale
HbA1c: (glycated) haemoglobin A1c
HRQL: Health-related quality of life
ICD-9-CM: The International Classification of Diseases, Ninth Revision, Clinical Modification
ICU: Intensive Care Unit
LDL: low-density lipoprotein
MRC: Medical Research Council [classification of heart failure]
NEADL: Nottingham Extended Activities of Daily Living Scale
NYHA: New York Heart Association [classification of heart failure]
OARS ADL: Older Americans Resources and Services Activities of Daily Living Scale
PHQ-9: Nine-item Patient Health Questionnaire
PIP-DCG: Principal Inpatient Diagnostic Cost Group Model for Medicare Risk Adjustment
QOF: Quality and Outcomes Framework
RAND-36: Research And Development Corporation 36-item health survey
RCT: Randomised controlled trial
RxRisk model: A revision and expansion of the Chronic Disease Score [33,55]
RxRiskV: Veterans Health Administration Adapted RxRisk
SCL-90-R: Symptom Checklist-90-Revised
SF-12: 12-item short-form health survey
SF-36: Medical Outcomes Study Short Form
WHO ATC: World Health Organisation Anatomical Therapeutic Chemical classification system

eTable 7: Development of models in original index descriptions

Publication and name of index	Method of developing model	Model details provided	Baseline outcomes reported
**Corrao 2017 [2]**<br>**Multisource Comorbidity Score**	Parametric survival models for relationship between each condition and time to death, then least absolute shrinkage and selection operator (LASSO) method to select predictor conditions, then coefficients multiplied by 10 and rounded to nearest integer	Regression coefficients of survival model for all included conditions. No intercepts or baseline survival	No mortality figures reported
**Wen 2017 [3]**<br>**Multimorbidity Frailty Index (mFI)**	Overall index score = number of conditions (“deficits”) divided by total candidate conditions. No weighting	Not applicable	30,136 deaths (35.0% sample). No figures for number of hospitalisations or intensive care admissions
**Stanley 2017 [4]**<br>**Measuring multimorbidity (M3) Index**	Conditions weighted by β coefficient where β>0. Total score = sum of β coefficients	All coefficients listed. No other model details	28,611 deaths (0.9% sample) in derivation and validation sets
**Wei 2016 [5]**<br>**Multimorbidity Weighted Index**	Used mixed models to predict physical function (PF) of SF-36 for each condition. Pooled coefficients from three samples using fixed-effects meta-analysis to develop condition weights	All coefficients listed. No other model details	PF used for weighting; no clear overall outcome. Summary PF scores given
**Robusto 2016 [6]**<br>**Drug-Derived Complexity Index**	Weights derived from Cox proportional hazard regression coefficients for mortality divided by 0.3, rounded to the nearest integer	All coefficients listed. No other model details	>213,000 deaths in combined samples (10.7%)
**Lorem 2016 [7]**<br>**Health Impact Index**	Weights from ordinal logistic regression odds ratios (odds of scoring at lower levels of self-rated health for those with the	Odds ratios for all components given. Full model equation given. No intercept	Summary statistics listed for self-rated health across demographic groups

Publication and name of index	Method of developing model	Model details provided	Baseline outcomes reported
	disease compared with those without), rounded to nearest integer		
**Pati 2016 [8] Multimorbidity Assessment Questionnaire for Primary Care (MAQ-PC)**	Not applicable	Not applicable	Not applicable
**Hong 2015 [9] estimated Physician Defined Complexity (ePDC)**	Logistic regression to identify factors predictive of physician-defined complexity (the odds of being defined as complex by physician)	Odds ratios for all components given. No intercept or further model details	Figures for all relevant outcome events given
**Wister 2015 [10]**	One model weighted by Health Utility Index (HUI3) correlation, one weighted by OARS functional status scale correlation, one weighted by β coefficients predicting HUI3 in ordinary least squares regression	All coefficients and correlations listed. No intercept or further model details	Summary statistics for all outcomes given
**Brettschneider 2013 [11]**	Conditions weighted by physician-defined severity	Not applicable	EQ-5D summary scores listed
**Min 2013 [12] Geriatric CompleXity of Care Index (GXI)**	Conditions weighted by physician-defined severity	Not applicable	Summary values for each outcome given
**Carey 2013 [13] QOF Comorbidity Score**	Conditions weighted by Cox proportional hazard ratios for mortality in training set	No coefficients or other model details	10,595 deaths (3.3% of sample)
**Dong 2013 [14] Pharmacy-Based Disease Indicator**	Drugs weighted based on coefficients from logistic regression for hospitalisation in training set	All coefficients listed. Equation given. No intercepts	Approximately 60,000 hospitalisations in each set (8.5% of sample)

Publication and name of index	Method of developing model	Model details provided	Baseline outcomes reported
**Bernabeu-Wittel 2011 [15] PROFUND**	Cox models for one-year mortality; weights generated by β coefficient divided by the lowest β coefficient and rounded to nearest integer. Components included if independently associated with mortality	Odds ratios (not coefficients) for each included component given. No intercepts	Mortality reported as 35% in derivation cohort (n≈265)
**Mukherjee 2011 [16] Health-Related Quality of Life Comorbidity Index (HRQL-CI)**	Predictors selected using LASSO method: regression for each predictor and either PCS or MCS of SF-12. Points generated based on coefficients and clinical judgement	All coefficients listed. Intercepts given for separate physical and mental SF-12 scores. Tested separately for coefficient- or points-based models	Summary statistics for outcomes presented
**Newman 2008 [17] Physiologic Index of Comorbidity**	Parameters weighted by arbitrary abnormality cut-points (tertiles)	Not applicable	No figures listed for number of deaths. Method of measuring disability not given. Summary scores for disability not given
**Tooth 2008 [18]**	Conditions weighted by regression coefficient (scaled and rounded to nearest integer) from Cox hazard models for mortality, logistic regression for healthcare outcomes, or multiple regression for SF-36. Different weights for each outcome	Hazard ratios and odds ratios for mortality and healthcare measures. Coefficients for all aspects of SF-36 listed. No intercept	14.9% of derivation set died (n≈777). Summary results given for all other outcomes
**George 2006 [19] Medication-based Disease Burden Index (MDBI)**	Weights based on association with disability in previous studies. Clinical panel decided on medications to include	None	Mortality or hospitalisation within 12 weeks (77 occurrences of one or both)
**Lee 2006 [20]**	Used backward elimination (*P*<0.05) to choose variables that improved predictive value of model, then further selected	Odds ratios but not coefficients given. No intercepts	1,361 (12%) deaths

Publication and name of index	Method of developing model	Model details provided	Baseline outcomes reported
	using Schwarz Bayesian Information Criterion (BIC). Weighting developed by dividing logistic regression β coefficients by the lowest coefficient and rounding to the nearest integer		
**Farley 2006 [21]**	Addition of various variables (e.g. count of physician visits and unique prescriptions). No weighting	Not applicable	Summary statistics for specific expenditure-related outcomes given
**Byles 2005 [22]**	Weights generated using hazard ratios for mortality and odds ratios for admission. Weights with and without health self-rating	Lists of odds and hazard ratios given but no coefficients or intercepts	51% admitted to hospital (485 patients), 7% died (n=59 in derivation sample, n=29 validation)
**Bayliss 2005 [23]**<br>**Disease Burden Morbidity Assessment**	Conditions weighted by self-reported severity	No; correlations between overall score and outcomes for testing only	Summary statistics for each outcome provided
**Selim 2004 [24]**	Components chosen by availability and expert panel. Weighting was not beneficial to model so was not used (data not shown in original paper)	Not applicable	77 patients (4%) died. No summary statistics for other outcomes
**Pope 2004 [25]**<br>**The Centers for Medicare and Medicaid Services' Hierarchical Condition Category (CMS-HCC)**	Developed using weighted least squares multiple regression	Coefficients for all parameters included. No intercepts	Detailed summary statistics of all expenditures given
**Sangha 2003 [26]**<br>**Self-Administered Comorbidity Questionnaire**	Conditions weighted by patient-reported severity	Not applicable	Summary statistics given for SF-36, hospitalisation and inpatient cost outcomes
**Fan 2002 [27]**<br>**Seattle Index of Co-morbidity**	Conditions weighted by regression coefficients from Cox hazard models for mortality,	Hazard ratios and coefficients for mortality for each parameter listed	396 (7.2% of sample) deaths, 1,383 (25.3%) hospitalisations

Publication and name of index	Method of developing model	Model details provided	Baseline outcomes reported
	multiplied by four then rounded to the nearest integer		
**Desai 2002 [28]**<br>**High-Risk Diagnoses for the Elderly Scale**	Conditions weighted by hazard ratio for one-year mortality, rounded to the nearest integer	Hazard ratios listed. No coefficients or intercepts	154 (29%) died within one-year follow-up
**Crabtree 2000 [29]**<br>**Comorbidity Symptom Scale (CmSS)**	Conditions weighted by self-reported severity	Not applicable	Appropriate scales used for each outcome but no summary statistics given
**Hornbrook 1996 [30]**	In one model, conditions weighted by interaction effects with RAND-36	Full model including intercepts given	No summary data given on healthcare cost outcomes
**Greenfield 1995 [31]**<br>**Total Illness Burden Index (TIBI)**	Conditions weighted by severity; each group's weight calculated by summing each condition's regression coefficients for functioning, combined with clinical opinion	Coefficients for one example condition included. No intercepts	Physical function and role functioning from SF-36. Summary data given
**Parkerson 1993 [32]**<br>**Duke Severity of Illness Checklist (DUSOI)**	Conditions weighted by severity for each patient	Not applicable	Not applicable
**Von Korff 1992 [33]**<br>**Chronic Disease Score**	Drugs weighted based on expert opinion	Not applicable	Summary data for outcomes in population validation cohort. 1,053 deaths (0.9%), 8,585 hospitalisations (8.0%)
**Starfield 1991 [34]**<br>**Ambulatory Care Groups**	Conditions classified into groups then treating as counts; no weighting	Not applicable	Summary measures for healthcare visits given
**Charlson 1987 [35]**<br>**Charlson Index**	Conditions weighted by adjusted relative risks for one-year mortality, rounded to nearest integer	Relative risks listed. No coefficients or intercepts	Mortality (in-hospital and at one year) figures listed for each condition

Publication and name of index	Method of developing model	Model details provided	Baseline outcomes reported
**Linn 1968 [36]**<br>**Cumulative Illness Rating Scale (CIRS)**	Conditions weighted by physician-defined severity	Not applicable	None measured in original paper

Abbreviations in eTable 7:

AUC: Area under the curve
BIC: Bayesian Information Criterion
CDS: Chronic Disease Score
EQ-5D: EuroQol five-dimension measure of health status
IDI: Integrated discrimination improvement
LASSO: Least absolute shrinkage and selection operator
MCS: Mental component summary
NRI: Net reclassification improvement
PCS: Physical component summary
PF: 10-item physical functioning scale of SF-36
ROC: Receiver operating characteristic
SF(-36 or -12): Medical Outcomes Study Short Form

eTable 8: Original indices that include aspects of mental health as comorbidities or outcome variables

First author and year	Comorbidities or index components	Outcomes	Findings, if applicable
**Corrao 2017 [44]**	Alcohol abuse, psychoses, anxiety and dementia included as comorbidities, defined by use of relevant medications	All-cause hospitalisation as outcome; no separation of admission types to include psychiatric	Regression coefficients with time to death: alcohol abuse β=0.99 (SE=0.16), psychoses β=0.77 (SE=0.05), anxiety β=0.52 (SE=0.23), dementia β=0.51 (SE=0.06). Weights allocated in model accordingly
**Wen 2017 [42]**	Comorbidities include “senile and presenile organic psychotic conditions.” Diagnoses from claims records	Does not specify subtypes of hospital admissions (e.g. psychiatric)	Not separately examined
**Stanley 2017 [49]**	Comorbidity variables: alcohol abuse, anxiety and behavioural disorders, dementia, drug abuse, major psychiatric disorder, mental and behavioural disorders due to brain damage, mental retardation. Diagnoses from routinely collected healthcare data	None	β coefficients for one-year mortality (log hazard ratios (95%CI)): alcohol abuse=0.58 (0.47 to 0.68), anxiety and behavioural disorders=0.12 (0.04 to 0.21), dementia=1.02 (0.97 to 1.07), drug abuse=0.56 (0.38 to 0.74), major psychiatric disorder=0.21 (0.13 to 0.29), mental and behavioural disorders due to brain damage=0.04 (−0.17 to 0.24), mental retardation=1.41 (1.21 to 1.60)
**Wei 2016 [17]**	Weighted comorbidities include alcohol abuse, depression and dementia. Taken from self-reported conditions	None	Depression and dementia both significantly associated with poorer scores on physical functioning subscale of SF-36
**Robusto 2016 [45]**	Drugs in model include anti-depressants, anti-psychotics, anti-dementia drugs. Data from record linkage	Does not specify whether hospitalisation outcome includes psychiatric admissions	Hazard ratios (95% CI) for mortality with each class: antipsychotics=2.32 (2.24 to 2.40), anti-dementia drugs=3.10 (2.92 to 3.29), antidepressants=1.09 (1.06 to 1.11). Weighted accordingly
**Pati 2016 [51]**	Comorbidities include dementia (self-report), depression (PHQ-9) and mental aspects of HRQL (SF-12)	None	Self-reported depression did not correlate well with PHQ-9 scores, suggesting under-diagnosis (no details given)
**Hong 2015 [18]**	Comorbidities include: anxiety, post-traumatic stress disorder, bipolar disorder, dementia, depression, drug or alcohol addiction-related conditions, personality disorder, schizophrenia or other psychotic disorders, chronic pain, eating disorders, or history of domestic violence, situational stress/depression/anxiety or adjustment reactions, attention deficit disorder,	None	High risk psychiatric and behavioural disorders were significantly associated with physician-defined complexity whereas post-traumatic stress disorder was not

First author and year	Comorbidities or index components	Outcomes	Findings, if applicable
	dementia, marijuana use, history of drug or alcohol addiction. Diagnoses from health records		
**Brettschneider 2013 [48]**	Depression, anxiety and somatoform disorders included as comorbidities in weighted count. Taken from self-report questionnaire. (Dementia was an exclusion criterion)	Anxiety/depression is one dimension of EQ-5D which was outcome	Weighted count of morbidities significantly associated with all domains of EQ-5D: b=-1.02 (SE 0.06). No information on weighted count's association with specific dimensions. Participants with depression had increased odds of poor scores across all EQ-5D domains. Depression, anxiety and insomnia were each significantly associated with EQ-5D anxiety/depression dimension
**Min 2013 [19]**	Dementia, anxiety and depression as comorbidity variables. Taken from medical records, weight assigned by expert consensus	None	Not examined separately
**Carey 2013 [40]**	Dementia, depression and psychotic disorders all included as comorbidities. Taken from medical records	None	All included mental disorders were individually associated with increased mortality risk in derivation set (dementia hazard ratio=2.83 (95% CI 2.63 to 3.04), depression HR 1.11 (1.05 to 1.18), psychotic disorders HR 1.74 (1.49 to 2.04))
**Dong 2013 [43]**	Psychiatric medication included as an index component	Does not specify whether outcome includes psychiatric hospitalisation	Weights in index: antidepressants=0.23, antipsychotics=0.40, lithium=0.85, anxiolytics=0.14. No specific outcomes given
**Bernabeu-Wittel 2011 [47]**	Dementia and delirium (in last hospital admission) as comorbidities in index. From records or self-report	None	Odds ratio for 12-month mortality with dementia=1.89 (95% CI 1.1 to 3.1), P=0.019, for delirium in last hospital admission=2.1 (1.5 to 4.9), P=0.001
**Mukherjee 2011 [20]**	Comorbidities include: affective disorders, schizophrenia, other psychoses, anxiety, depression. Diagnoses from medical records. Cognitive impairment codes excluded	SF-12 mental component score, two core health status questions	Mental health diagnoses had strong association with worse SF-12 MCS scores, as did asthma, heart failure, neurological conditions and pain-related conditions
**Tooth 2008 [38]**	Depression, anxiety, Alzheimer's disease (self-reported)	SF-36 as outcome, includes mental component scale. Healthcare visits (self-reported) as outcome – does not specify which specialty	Alzheimer's disease found to be associated with higher risk of mortality, functional dependency and poorer social functioning and mental health. Depression had a weak link with poor functional ability

First author and year	Comorbidities or index components	Outcomes	Findings, if applicable
**George 2006 [39]**	Alzheimer's and other dementias included as comorbidity (identified by prescription of drugs for dementia)	Does not specify whether outcome includes psychiatric hospitalisation	MDBI reported as 100% sensitive and 100% specific for Alzheimer's and other dementias when measured against medical records
**Lee 2006 [22]**	Two functional questions (difficulty managing finances and personal hygiene) refer to "health or memory problems"	None	Difficulty bathing and managing finances each assigned two points in overall model
**Byles 2005 [37]**	Depression and forgetfulness included as comorbidities. Self-reported with severity rating	Mental Component Score (MCS) of SF-36	Increasing scores on all versions of the multimorbidity index were associated with worse scores on the SF-36 MCS
**Bayliss 2005 [24]**	None	Depression screen from Behavioural Risk Factor Surveillance System	Being less depressed was significantly negatively correlated with self-reported disease burden (P<0.001) and number of conditions (P=0.002) but not with Charlson index or RxRisk score
**Selim 2004 [25]**	Comorbidity variables include self-reported schizophrenia, depression, bipolar disorder, anxiety disorder, post-traumatic stress disorder, alcohol abuse	Mental health outpatient visits from administrative data, SF-36	Mental disorders on comorbidity index correlated better with the mental than physical scale of the SF-36.<br>Comorbidity index including mental disorders was not significantly associated with mortality
**Pope 2004 [26]**	Comorbidity variables: Drug or alcohol psychosis, drug or alcohol dependence, schizophrenia, major depressive, bipolar, and paranoid disorders. Diagnoses taken from claims data	None	Not separately examined
**Sangha 2003 [27]**	Depression (self-reported diagnosis) included as a comorbidity	Mental Component Score (MCS) of SF-36	Spearman correlation of SF-36 MCS score at one-year follow-up with baseline comorbidity score R_2=-0.03 (P>0.05).
**Crabtree 2000 [41]**	Anxiety/depression (self-report) included as one comorbidity	Anxiety/depression measured by Hospital Anxiety and Depression (HAD) scale and GHQ-28	Overall score on the CmSS correlated with GHQ-28 (*r*=0.48) and HAD (*r*=0.52) with P<0.01

First author and year	Comorbidities or index components	Outcomes	Findings, if applicable
**Hornbrook 1996 [30]**	Depression as comorbidity (self-report diagnosis); some models included elements of SF-36, which includes mental health	None	Depression performed poorly at explaining variance in costs, both as a reported comorbidity and through SF-36
**Greenfield 1995 [31]**	Not clearly included as comorbidity (may be counted under "neurologic problems")	"Mental health index" (assume part of SF-36)	Global severity measure significantly associated with mental health index score (F statistic 51.7, P<0.001)
**Parkerson 1993 [32]**	Any condition could be a comorbidity (Diagnoses from medical records, weighted by rater's clinical judgement)	None	Participants with the highest scores were those with depression and at least one other condition
**Von Korff 1992 [33]**	Psychotropic drugs not included	Depression, anxiety, somatisation as outcomes in one test (symptoms measured with SCL-90-R)	Chronic Disease Score was not correlated with depression and anxiety
**Starfield 1991 [34]**	Three of 34 listed diagnoses are psychosocial, separated into chronic, other and psychophysiologic. Taken from health records	Does not specify subtype of outpatient visits (e.g. mental health	Individuals with psychosocial diagnoses, whether alone or in combination with other diagnoses, have relatively high levels of healthcare use
**Charlson 1987 [35]**	Dementia, according to medical records, included as comorbidity	None	Dementia alone carried relative risk of one-year mortality of 1.4
**Linn 1968 [36]**	Psychiatric disease listed as disease area (severity scored by physician)	None	Not examined separately

eTable 9: Usage, validation and performance of multimorbidity indices – indices without external validation[1]

Publication and name of index	Citations since publication[2]	Citations per year	Internal validation and/or comparison	Predictive accuracy measurement	Performance
**Corrao 2017 [2] Multisource Comorbidity Score**	9	9.0	Tested in original paper on one internal (split-sample) and three external validation sets. Compared to Charlson, Elixhauser and Chronic Disease Score (CDS) indices	Discrimination: AUC for one-year mortality	AUCs for one-year mortality: Multisource Comorbidity Score=0.78 (95% CI 0.77 to 0.79), Charlson=0.69 (0.68 to 0.70), Elixhauser=0.65 (0.64 to 0.66), CDS=0.69 (0.68 to 0.70)
**Wen 2017 [3] Multimorbidity Frailty Index (mFI)**	8	8.0	ROC analysis of outcomes within original dataset only	Discrimination: AUC for all outcomes by categorised mFI scores	C-statistics for: all-cause mortality=0.67 (95% CI 0.66 to 0.68), unplanned hospitalisation=0.65 (0.65 to 0.66) and ICU admission=0.68 (0.67 to 0.69) (all at one year)
**Stanley 2017 [4] Measuring Multimorbidity (M3) Index**	12	12.0	Validated in original paper on validation set (randomly assigned split-sample). Compared with Charlson and Elixhauser indices	Discrimination: C-statistics for mortality and hospitalisation (also used integrated discrimination improvement (IDI) and Akaike information criterion)	C-statistics for one-year mortality: M3 + age + gender=0.92 (95% CI 0.93 to 0.93), Charlson + age + gender=0.92 (0.92 to 0.92), Elixhauser + age + gender=0.92 (0.92 to 0.93); One-year hospitalisation: M3 + age + gender=0.70 (95% CI 0.70 to 0.71), Charlson + age + gender=0.68 (0.68 to 0.69), Elixhauser + age + gender=0.68 (0.67 to 0.68)
**Robusto 2016 [6] Drug-Derived Complexity Index (DDCI)**	10	5.0	Validated within original paper on validation cohort (randomly assigned split-sample). Compared with Charlson index in subsample of 125,094 hospitalised patients	Discrimination and net reclassification improvement (NRI) measured for mortality and hospitalisation	C-statistics for one-year mortality: DDCI=0.81 (0.81 to 0.82), age, sex and Charlson combined=0.80 (0.79 to 0.80); overall mortality: DDCI=0.80 (0.79 to 0.80), age, sex and Charlson combined=0.79 (0.78 to 0.79); first unplanned hospitalisation: DDCI=0.62 (0.62 to 0.62), age, sex and Charlson combined=0.62 (0.62 to 0.62)
**Lorem 2016 [7] Health Impact Index (HII)**	11	5.5	Validated in original paper in separate cohort. Compared ability to predict self-	Calibration: used Spearman's correlation to compare the association of self-rated health to both Health Impact Index	Spearman correlation (R_S) with self-rated health: HII= -0.36, *P*<0.001, Charlson= -0.25, *P*<0.001

1 Validation of original indices only; validation of index updates not included. Papers marked with * have updates available
2 Citations per full year since publication according to Google Scholar, as at 7th September 2019

Publication and name of index	Citations since publication2	Citations per year	Internal validation and/or comparison	Predictive accuracy measurement	Performance
			rated health with Charlson index	and the Charlson index (which was originally developed to predict mortality)	
**Pati 2016 [8] Multimorbidity Assessment Questionnaire for Primary Care (MAQ-PC)**	7	3.5	Tested for internal consistency within original cohort. Comparison between patient self-report of diagnoses and physicians' prescriptions. Not compared with other scales	Compared self-report with diagnoses apparent from prescriptions	Overall Cronbach's alpha=0.69. Concordance (Scott Kappa) between self-report and prescription-based diagnoses ranged from 0.58 for hearing problem to 1.00 for tuberculosis
**Hong 2015 [9] estimated Physician Defined Complexity (ePDC)**	15	5.0	Test characteristics calculated in validation set within original paper. Bootstrapping in random third of sample. Compared with outpatient Charlson score and proprietary commercial risk predictor, but no comparable prediction scores given	Discrimination: C-statistics for physician-defined complexity. Compared to Charlson and Commercial Risk Predictor, but no clear comparison results available	In own validation set: Accuracy=0.82, Sensitivity=0.47, Specificity=0.95, Positive Predictive Value=0.77, Negative Predictive Value=0.83. C-statistics for models' prediction of physician-defined complexity: <45 years=0.82, 45–64 years=0.82 and ⩾65 years=0.77
**Wister 2015 [10]**	15	5.0	Measured construct validity of six models with each other within original paper	None	Bivariate correlation coefficient for multimorbidity additive scale (best performing model) with life satisfaction= -0.23 (compared to dichotomised 0/⩾1 conditions= -0.10), perceived health= -0.39 (-0.15), health professional visits= 0.22 (0.14), daily medication use= 0.50 (0.30)
**Brettschneider 2013 [11]**	85	17.0	None	None	Ordinary least squares regression for association between multimorbidity measured by weighted count score with overall HRQL (EQ VAS), b= -1.02 (SE=0.06)

Publication and name of index	Citations since publication[2]	Citations per year	Internal validation and/or comparison	Predictive accuracy measurement	Performance
**Min 2013 [12] Geriatric CompleXity of Care Index (GXI)**	26	5.2	In original paper, measured correlation and compared predictive ability with simple disease count, modified Charlson index (mCCI) and Hierarchical Condition Category score (mHCC) in same cohort	Discrimination: AUC for all outcomes compared to modified Charlson index (mCCI) and modified HCC (mHCC)	Adjusted R^2s for primary care visits: GXI=14.3, mHCC=6.1, mCCI=3.8; specialty visits: GXI=9.1, mHCC=9.9, mCCI=2.9; quality indicators (medical records only): GXI=32.8, mHCC=15.8, mCCI=19.6. Adjusted AUCs for 5-year mortality: GXI=76.2, mHCC=78.7, mCCI=77.5; 5-year functional decline: GXI=83.8, mHCC=89.6, mCCI=77.5; Polypharmacy (⩾14 medications): GXI=81.5, mHCC=76.9, mCCI=70.1
**Carey 2013 [13] QOF Comorbidity Score**	44	8.8	Tested within original paper on validation cohort (split-sample); also compared mortality prediction with Charlson index	Discrimination: C-statistics for mortality compared to Charlson index	C-statistics for one-year mortality: standard QOF score=0.83, extended QOF score=0.83, Charlson index=0.82
**Newman 2008 [17] Physiologic Index of Comorbidity * (PIC)**	98	9.8	Compared with age alone and simple condition count within same cohort in original paper	Discrimination: survival models by index score	AUCs for nine-year mortality in original paper: age alone=0.67, PIC=0.71, PIC adjusted for age, sex, race=0.73
**Tooth 2008 [18]**	86	8.6	Validated within original paper on validation set (random split-sample)	Explained variation: compared R^2 of weighted and unweighted scores for individual conditions across 13 analyses	Relative differences in R^2 for weighted scores: 0.2-1.3% (median=0.9%), unweighted scores 4.9%-35.0%, median=13.3%
**Farley 2006 [21]**	167	13.9	Compared with Charlson-Romano index, Elixhauser index and RxRisk-V in same cohort in original paper	Discrimination: C-statistic for individuals spending at the 90th percentile for each model	C-statistics for individuals spending at 90th percentile on hospital and physician claims: Farley diagnosis clustering=0.69 (0.68 to 0.70), Charlson=0.66 (0.65 to 0.67), RxRisk-V 0.64 (0.63 to 0.65), Elixhauser=0.66 (0.66 to 0.66)
**Byles 2005 [22]**	94	7.2	Tested different models on validation cohort within original paper (random split-sample)	None	HRs for two-year mortality using severity-weighted index based on mortality=1.3 (*P*<0.001), severity-weighted index based on hospitalisation=1.1 (*P*>0.05). ORs for two-year hospitalisation using severity-weighted index based on mortality=1.2 (*P*>0.05), severity-weighted index based on hospitalisation=1.7 (*P*<0.01).

Publication and name of index	Citations since publication[2]	Citations per year	Internal validation and/or comparison	Predictive accuracy measurement	Performance
					Authors advise that a single index cannot predict a variety of outcomes
**Selim 2004 [24]**	191	13.6	Compared one model to Disease Burden Index (DBI) in same cohort in original paper [37]	Linear regression for variance in outcomes explained by index models. No discrimination or calibration	Pearson correlations: combined physical/mental comorbidity model with: SF-36 PCS= -0.39, SF-36 MCS= -0.31. Condition/symptom comorbidity index with: PCS= -0.50, MCS= -0.39. Hazard ratio for 35-week survival with each additional unit in physical index= 0.14. For MCS, a) R_2 for regression models: DBI=15%, combined physical + mental index=33%; b) regression coefficients: DBI= −1.32, combined physical + mental index= −5.50 (P<0.001). For psychiatric outpatient clinic visits, R_2: DBI=2.3%, combined physical + mental index=14%; coefficients DBI=0.06, Combined index=0.40; (P<0.001)
**Fan 2002 [27] Seattle Index of Co-morbidity (SIC)**	160	10.0	Tested within original paper on validation cohort (random split-sample)	Discrimination: ROC and Kaplan-Meier curves for mortality and hospitalisation	In validation set for a) two-year survival, AUCs: SIC=0.71, combined PCS+MCS= 0.71; b) two-year hospitalisations, AUCs: SIC=0.61, PCS+MCS=0.64
**Desai 2002 [28] High-Risk Diagnoses for the Elderly Scale * (HRDES)**	85	5.3	Validated within original paper; compared with Charlson-Deyo index and APR-DRGs in separate validation cohort	Discrimination: Kaplan-Meier curves for mortality by score risk level	For one-year mortality in validation cohort, C-statistics: HRDES=0.69, Charlson-Deyo=0.65 (P<0.05 compared to HRDES), total diagnoses=0.59 (P<0.05 compared to HRDES), APR-DRGs=0.67 (P=0.43 compared to HRDES)
**Crabtree 2000 [29] Comorbidity Symptom Scale (CmSS)**	55	3.1	Correlations with outcomes tested in separate validation set in original paper. Not compared to another scale	None	Spearman's coefficient for correlation between CmSS score and activities of daily living (NEADL)=0.56; perceived health status (GHQ-28)=0.48; anxiety and depression (HAD)=0.52 (P<0.01 for all values)
**Hornbrook 1996 [30]**	168	7.6	Models compared with each other on half of the sample in original paper (random split-sample). Not compared to other scales	Calibration: regression of predicted versus actual costs	Grouped R_2s for fit of predicted to actual costs: Disease count=0.56, Disease count + age + gender =0.68, Disease count, age, gender and function=0.80

Publication and name of index	Citations since publication[2]	Citations per year	Internal validation and/or comparison	Predictive accuracy measurement	Performance
**Greenfield 1995 [31] Total Illness Burden Index (TIBI)***	133	5.8	Correlations with outcomes tested in single cohort in original paper. Not compared with any other scales	None	Pearson's *r* for correlation with global severity measure: physical function= -0.55 (*P*<0.001); role functioning due to physical health= -0.54 (*P*<0.001); log(disability days)=0.43 (*P*<0.001); log(physician visits)=0.28 (*P*<0.001); log(hospitalisations)=0.15 (*P*<0.001)

eTable 10: Usage, validation and performance of multimorbidity indices – Indices with external validation

Publication and name of index	Citations since publication[3]	Citations per year	Validation and/or comparison	Predictive accuracy measurement in original paper	Performance (original outcomes)	Additional outcomes tested in external validation
**Wei 2016 [5] Multimorbidity Weighted Index (MWI)**	22	11.0	Internally validated through bootstrapping and cross-validation to test weighting. Tested for specific outcomes in follow-up paper by same author group.[38] Not compared to another model	None	Externally validated in 20,509 participants of the Health and Retirement Survey. Mean (SD) age 64.7 (10.7) years, 54.1% women. On multivariable regression, adjusted β for each point increase in MWI: physical functioning score= -3.73 (95% CI -3.84 to -3.62), grip strength= -0.27kg (-0.32 to -0.22), gait speed= -0.29m/s (-0.35 to -0.23), TICS-m score= -0.06 (-0.07 to -0.04) [38]	None
**Dong 2013 [14] Pharmacy-Based Disease Indicator (PBDI)**	18	3.6	Prediction of hospitalisation compared with Charlson-Deyo index in separate sample in original paper. Compared to medication and condition counts in subsequent paper by different authors.[39]	Discrimination: C-statistics and IDI. Calibration: Brier score. Compared performance with Charlson-Deyo using net reclassification improvement (NRI)	C-statistics for one-year hospitalisation: a) in original paper: PBDI=0.72 (adjusted for age + sex), Charlson-Deyo=0.69 (adjusted for age + sex).[14] b) In validation paper (449,715 French workers, approximately 52% female): PBDI=0.68 (adjusted for age + gender), adjusted condition count=0.64, alternative medication index (Individual Chronic Condition score)=0.68 [39]	One-year mortality. C-statistics for one-mortality PBDI=0.90 (adjusted for age + gender), adjusted condition count=0.90, alternative medication index (Individual Chronic Condition score)=0.89 [39]
**Bernabeu-Wittel 2011 [15] PROFUND**	133	19.0	Compared with Charlson-Deyo index (with and without age adjustment) in separate validation cohort	Calibration: compared index-predicted to observed mortality. Discrimination: ROC curves for final model in	AUCs (95% CI) for one-year mortality in original paper: PROFUND=0.73 (0.71 to 0.76), Age-adjusted Charlson-Deyo=0.62 (0.59 to 0.65).[15] AUCs for four-year mortality: a) in 768 people with multimorbidity (45.3% women, mean (SD) age 78.8 (9.8) years)	Unplanned hospitalisation. In 1,033 cardiology inpatients, mean (SD) age 67 (13.1) years 35.1% women, HR for mortality=1.13 (1.01-1.27) and either mortality

[3] Citations per full year since publication according to Google Scholar, as at 7th September 2019

Publication and name of index	Citations since publication[3]	Citations per year	Validation and/or comparison	Predictive accuracy measurement in original paper	Performance (original outcomes)	Additional outcomes tested in external validation
			in original paper. Validated in four subsequent papers, three of which included an original author.[40–42]	derivation and validation sets	PROFUND=0.71 (0.67 to 0.77), Age-adjusted Charlson-Deyo=0.61 (0.56 to 0.67) [40] b) in 441 people with multimorbidity (mean (SD) age 80.9 (8.7) years, 55.6% women) [41] PROFUND in internal medicine=0.75 (0.69-0.81), Geriatric medicine=0.52 (0.37 to 0.67). C-statistic for one-year mortality in 333 internal medicine patients with multimorbidity (mean (SD) age 79.3 (9.0) years, 50.3% women)=0.73 (0.67 to 0.78), 132 geriatric medicine patients with multimorbidity (mean (SD) age 84.6 (7.1) years, 65.0% female)=0.55 (0.45 to 0.64) [42]	or hospitalisation=1.09 (1.01-1.18), both at 12 months.[43]
**Mukherjee 2011 [16] Health-Related Quality of Life Comorbidity Index (HRQL-CI) ***	32	4.6	Internal validation with 10-fold cross-validation. Compared with Charlson index in separate validation cohort in original paper. Validated in two subsequent papers, one by separate authors [44]	Calibration: correlation between observed and predicted MCS or PCS	Correlation between observed and predicted a) PCS: HRQL-CI=0.57, Charlson=0.38 b) MCS: HRQL-CI=0.37, Charlson=0.11. Discrimination of multiple outcomes assessed among 9,832 patients with type 2 diabetes (mean (SD) age 44.8 (11.6) years, 73.1% female) in external validation paper.[45] HRQL-CI physical=0.66 (0.65 to 0.68), HRQL-CI mental=0.66 (0.64 to 0.68). In 13,289 adults with diabetes (mean (SD) age 60.5 (13.7) years, 49.0% female), adjusted R_2 for predicting a) PCS: HRQL-CI=29.1 (27.5 to 30.6), Charlson=19.1 (17.6 to 20.5), Elixhauser=21.1 (19.6 to 22.4), CDS=26.3 (24.7 to 27.7) b) MCS: HRQL-CI=15.0 (14.3 to 17.4), Charlson=5.6 (4.7 to 6.6), Elixhauser=14.3 (12.8 to 15.8), CDS=14.7 (13.3 to 16.2) [44]	Healthcare costs, medication adherence, hospitalisation and outpatient attendance. C-statistics for healthcare costs: Charlson=0.64 (95% CI 0.62 to 0.66), Elixhauser=0.70 (0.68 to 0.71), CDS=0.65 (0.64 to -0.67).[45]
**George 2006 [19] Medication-based Disease Burden Index (MDBI)**	44	3.7	Compared with Chronic Disease Score and Charlson index on single cohort in original paper.	Tested predictive validity using odds ratios for outcomes	At predicting death and hospital readmission, ORs (95% CI): MDBI=4.7 (1.4 to 15.5), CDS=1.13 (1.0 to 1.3), Charlson=1.4 (1.2 to 1.7). In external validation on 212 acute geriatric inpatients, mean (SD) age 81 (7.3) years, 62%	Self-rated health. Reported as a statistically significant association between MDBI scores and decreasing self-rated health (*P*<0.001) [47]

Publication and name of index	Citations since publication3	Citations per year	Validation and/or comparison	Predictive accuracy measurement in original paper	Performance (original outcomes)	Additional outcomes tested in external validation
			Later validation papers by different authors compared to CIRS-G, Charlson and condition count [46] and tested association with mortality and self-rated health [47]		female, prediction of three-month mortality or readmission: MDBI=2.99 (0.99 to 9.03), CIRS-G=1.2 (1.1 to 1.3), Charlson index=1.39 (1.12 to 1.72), chronic disease count=1.22 (1.08 to 1.38).[46]<br>In 776 cohort study participants (mean age 83.5 years, 58% female), adjusted HR for mortality=3.69 (95% CI 2.26-6.02) [47]	
**Lee 2006 [20] ***	656	54.7	Tested within original paper on separate validation cohort and by original authors in ten-year follow-up.[48]<br>Subsequent validation by different authors compared to Charlson index [49]	Calibration: compared predicted with actual mortality in derivation and validation cohorts.<br>Discrimination: ROC curves in derivation and validation cohorts	C-statistics for four-year mortality: development cohort=0.84, validation cohort=0.82.<br>In follow-up of original participants for predicting ten-year mortality, C-statistic (validation cohort)=0.83 (0.82 to 0.84) [48]<br>External validation tested ten-year mortality in 735 patients undergoing radical cystectomy (median age 67 years): HR (95% CI) per unit increase in indices: Lee=1.06 (1.00 to 1.12, *P*=0.04), age-adjusted Charlson=1.08 (1.02 to 1.15, *P*=0.01).[49]	None
**Bayliss 2005 [23] Disease Burden Morbidity Assessment * (DBMA)**	200	15.4	Compared survey results with Charlson and RxRisk indices in single cohort in original paper.<br>Validity tested in three subsequent papers by different authors [50–52]	Discrimination: C-statistics for self-reported diseases only, not overall score	For overall health status score as outcome in original paper, Spearman correlations DBMA=0.60 (*P*<0.001), Charlson=0.48 (*P*<0.001), RxRisk=0.17 (*P*=0.037).[23] In subsequent paper (307 participants, mean age 59 years), Cronbach's alpha for internal consistency of the total DBMA score=0.69.[50]<br>Subsequent validity paper created a linear measure in 1,747 adults aged over 50 years. Spearman's correlations with: physical functioning= −0.48, perceived health=−0.47,	Mortality.<br>Five-year mortality validation in 625 community-dwelling adults aged ≥65 years for higher compared to lower DBMA scores HR=1.07 (95% CI 1.00–1.15, *P*=0.044) [51]

Publication and name of index	Citations since publication3	Citations per year	Validation and/or comparison	Predictive accuracy measurement in original paper	Performance (original outcomes)	Additional outcomes tested in external validation
					depression (score ≥3 on CES-D-10)=0.32, quality of life −0.24 [52]	
**Pope 2004 [25] The Centers for Medicare and Medicaid Services' Hierarchical Condition Category (CMS-HCC) ***	731	52.2	In single cohort in original paper, compared to risk adjustment model PIP-DCG. Evaluated predicted versus actual costs in subsequent papers by external groups [53,54] and report by original authors [55]	Calibration: Tests of predicted versus actual costs; predictive ratios given	In original paper, R_2 for one-year expenditure: age-sex model 1.0%, PIP-DCG 6.2%, DCG/HCC 11.2%.[25]<br><br>Among 1,441,247 Medicare beneficiaries, ratio of predicted to actual healthcare costs in all age and gender groups=1.000 [55]	Mortality.<br>In 170,342 patients admitted to hospital (mean age 78 years, 60% female), C-statistics for six-month mortality: CMS-HCC=0.72 (*P*>0.05), Charlson=0.71 (*P*<0.05), Elixhauser=0.70 (*P*<0.05) [53]<br>Hospitalisation<br>In 83,187 managed care patients with mean age 46.9 years, 54.6% female, c-statistic for predicting hospitalisation=0.67, emergency visits=0.58 [54]
**Sangha 2003 [26] Self-Administered Comorbidity Questionnaire * (SCQ)**	966	64.4	In single sample in original paper, tested correlation with Charlson index. Validated in two later papers by different authors [56,57]	None	In original paper, Spearman coefficients for a) correlation between SCQ and Charlson index=0.32 (0.55 when truncating to contain only comparable items); b) number of prescriptions at one year, SCQ=0.37, Charlson=0.02; c) frequency of doctor visits: SCQ=0.15, Charlson=0.09. R_2s for a) PCS at one year: Charlson="non-significant", SCQ=0.22 (69.3% variation explained by comorbidity); b) MCS: SCQ and Charlson both "non-significant". [26]<br>In external validation of 525 patients after acute coronary syndrome (mean (SD) age 59.7 (12.0) years, 36.4% female), R_2 for EQ-5D scores at eight months: Charlson=0.25 (*P*=0.132), SCQ=0.27 (*P*<0.001); Activity	None

Publication and name of index	Citations since publication[3]	Citations per year	Validation and/or comparison	Predictive accuracy measurement in original paper	Performance (original outcomes)	Additional outcomes tested in external validation
					Status Index, a measure of physical function, at eight months: CCI=0.37 (*P*<0.001), SCQ=0.36 (*P*<0.001).[56] In 98 outpatients with ankylosing spondylitis (mean (SD) age 53.9 (11.4) years, 29.6% female), Spearman coefficients for a) correlation between SCQ and Charlson=0.24; b) PCS: SCQ= -0.45, Charlson= -0.17; b) MCS: SCQ= -0.10, Charlson=0.09 [57]	
**Parkerson 1993 [32] Duke Severity of Illness Checklist (DUSOI)***	193	7.7	Comparison between clinicians' ratings and auditor for inter-rater reliability in single sample in original paper. Not compared to another scale. Prediction of one-year healthcare usage tested in later paper by same authors [58]	None	Intraclass correlation coefficients (ICC) of agreement for provider-completed analogue scale of overall illness severity for: DUSOI provider overall severity scores=0.61 (*P*<0.001); DUSOI audit checklist scores=0.42 (*P*<0.001). External validation tested inter-rater reliability in 14 sets of records by 33 clinicians [59]: ICC=0.43 (95% CI 0.27 to 0.61).	Healthcare usage. In 1,202 primary care patients (mean (SD) age 47.6 (16.6) years, 65.0% female), adjusted R^2 variance explained by DUSOI in all healthcare visits =0.05.[58]
**Von Korff [33] Chronic Disease Score***	942	36.2	Compared with physician rated severity scale in pilot sample and separate random sample. Compared with ADGs for cost prediction in later paper by the same authors [60]	None	In original paper, Pearson correlation between CDS and physician-rated physical disease severity in pilot sample (n=219) *r*=0.57, in second sample (n=722) *r*=0.46.[33] Also used as comparator when developing several other indices [2,16,19]	Healthcare costs. In later paper examining 254,694 managed care enrolees (no demographics available), ordinary least squares regression R^2s explaining variance in six-month total cost for age and sex=0.02, CDS adjusted for age and sex=0.09, ADGs adjusted for age and sex=0.19 and revised CDS (different

Publication and name of index	Citations since publication3	Citations per year	Validation and/or comparison	Predictive accuracy measurement in original paper	Performance (original outcomes)	Additional outcomes tested in external validation
						weighting method) adjusted for age and sex=0.19 [60]
**Starfield 1991 [34] Ambulatory Care Groups***	574	21.3	In original paper, compared distribution of diagnostic groups in subsamples from across five healthcare providers. Several external validations by different authors testing cost prediction, healthcare use and mortality [61]	None	In original paper, for predicting one-year total costs, adjusted R^2s: age group + sex=0.04, age group, sex, binary ADG=0.19, 51 ACGs=0.15.[34] Multiple external reviews in different settings test its predictive value.[61] In one cohort of 59,384 Medicaid members (no demographics given), AUC for 90th percentile of costs: Chronic illness and disability payment system (CDPS)=0.69 (95% CI 0.67 to 0.70), diagnostic cost groups (DCG)=0.75 (0.74 to 0.76), ACG-PM version 7.0 (adjusted clinical groups- predictive model [an adaptation])=0.79 (0.78 to 0.80).[62]	Mortality. In population of 10,498,413 adults (median age 46 (IQR 34-59) years, 51% women), one-year mortality C-statistics: age + sex=0.88, Charlson=0.91, 32 ADGs=0.87, age, sex + 32 ADGs=0.92 [63]
**Charlson 1987 [35] Charlson Index***	29408	948.6	Tested within original paper on separate validation cohort. Compared with Kaplan-Feinstein method. Extensively validated and used elsewhere by different authors, often as comparator when generating new indices	Discrimination: Kaplan-Meier plots of mortality with differing index levels compared to Kaplan-Feinstein method	Within original paper, RR for one-year mortality for: increasing Charlson index by 1 point=2.3 (95% CI 1.9-2.8), each decade of age=2.4 (2.0-2.9) In survival analysis, variance explained: Charlson=0.41, Kaplan-Feinstein=0.41.[35] Also used as comparator for many other indices listed in this table [1,3,4,5,7,10,11,32,35,40,41,47,49,60]	Several other outcomes tested including HRQL, medication use, length of hospital stay, readmission [64]

Publication and name of index	Citations since publication3	Citations per year	Validation and/or comparison	Predictive accuracy measurement in original paper	Performance (original outcomes)	Additional outcomes tested in external validation
**Linn 1968 [36] Cumulative Illness Rating Scale (CIRS) ***	1863	37.3	Validation briefly mentioned in original paper. Externally validated in several places by different authors [65–68]	None	Original study reports that "total scores correlated with death, vital organ involvement, number of previous illnesses at P<0.01" (but not age) with no further details.[36]	In study of 181 geriatric inpatients (mean (SD) age 79 (7.4) years), correlations between original CIRS and activities of daily living *r*=-0.49 (*P*<0.001), patient morale= -0.30 (*P*<0.001), days in hospital=0.21 (*P*=0.001), number of medications=0.31 (*P*<0.001).[65] In comparison of 238 adults in primary care (mean (SD) age 59.0 (14.3) years, 71.0% female), Pearson correlation coefficients for PCS: CIRS= -0.54 (*P*<0.01), Charlson= -0.31 (*P*<0.01).[68] In 103 inpatients aged 90-99 years (mean age 92 years, 71% female), CIRS correlated with length of hospital stay, Pearson's *r*=0.4, *P*<0.05.[67] Among 439 residents of a care facility with mean (SD) age 84.1 (5.7) years, 72.4% female, CIRS correlated with functional disability according to Physical Self-Maintenance Scale *r*=0.322 (*P*=0.001) and

Publication and name of index	Citations since publication3	Citations per year	Validation and/or comparison	Predictive accuracy measurement in original paper	Performance (original outcomes)	Additional outcomes tested in external validation
						total number of medications (*r*=0.301).[66]

* Indices that have an updated or modified version available

Abbreviations in eTables 9 and 10:
APR-DRGs: All Patient Refined Diagnosis Related Groups
AUC: Area under the Curve
CDS: Chronic Disease Score
CES-D: Center for Epidemiologic Studies Depression scale
CI: Confidence interval
CIRS-G: Cumulative Illness Rating Scale – Geriatric
EQ 5D: EuroQol five-dimension measure of health status
EQ VAS: EuroQol-visual analogue scale
HR: Hazard ratio
HRQL: Health-related quality of life
IDI: Integrated discrimination improvement
mFI: Multimorbidity Frailty Index
mHCC: Modified Hierarchical Condition Categories
mCCI: Modified Charlson Comorbidity Index
MCS: Mental Component Score of SF-36
NEADL: Nottingham Extended Activities of Daily Living Scale
NRI: Net reclassification improvement
OR: Odds ratio
PCS: Physical Component Score of SF-36
PIP-DCG: Principal Inpatient Diagnostic Cost Group Model for Medicare Risk Adjustment
QOF: Quality and Outcomes Framework
ROC: Receiver Operating Characteristic
RR: Risk ratio
RxRisk: A revision and expansion of the Chronic Disease Score (with and without Veterans' adaptation)
TICS-m: Modified Telephone Interview for Cognitive Status

eTable 11: Index updates and adaptations

Publication and name of index	Updates or adaptations	Aim compared to original index	Details	Performance or comparison
**Mukherjee 2011 [16] Health-Related Quality of Life Comorbidity Index (HRQL-CI)**	Ou 2016 [69]	Measuring health-related quality of life (same as original)	Combines physical and mental subscales of original measure into one scale	Regression coefficients for a) general health: refined HRQL-CI=0.25, Charlson=0.10 b) SF-6D: refined HRQL-CI=0.25, Charlson=0.09; c) EQ-5D: refined HRQL-CI=0.28, Charlson=0.06
**Newman 2008 [17] Physiologic Index of Comorbidity**	Modified Physiologic Index [70]	Predicting mortality (same as original)	Adapted to include more easily available measures in epidemiological studies. Parameters: systolic blood pressure, forced vital capacity, Digit Symbol Substitution Test score, serum cystatin-C, serum fasting glucose	C-statistics for mortality (mean follow-up 9.3 years): Unadjusted index=0.66 (95% CI 0.64 to 0.68), Age alone=0.59 (0.57 to 0.61), Index + age=0.67 (0.65 to 0.69)
	Healthy Aging Index [71]	Predicting mortality (same as original)	Adapted for use in epidemiological studies. Components include: systolic blood pressure, pulmonary vital capacity, creatinine, fasting glucose, and Modified Mini-Mental Status Examination (MMSE) score	C-statistics for mortality (median follow-up 12.8 years): index alone=0.64 (0.63 to 0.66), age alone=0.70 (0.68 to 1.72)
**Lee 2006 [20]**	Kobayashi 2016 [72]	Predicting ten-year mortality (compared to four in original index)	Weighted to predict ten-year mortality. Assigns different weights to age, includes a variable on lack of physical activity. Excludes some variables from original index (diabetes, BMI<25 kg/m_2, difficulty bathing)	AUC for ten-year mortality in validation cohort, with new index=0.84, with original index=0.81
**Bayliss 2005 [23] Disease Burden Morbidity Assessment**	Bayliss 2009 [73]	Predicting general health/disease burden (same as original)	Included 21 conditions instead of original 25 (excludes liver disease, kidney disease, alcoholism, nerve conditions)	Correlation coefficients with Charlson-Quan=0.23 (*P*<0.001) and CDS=0.26 (*P*<0.001)
	Poitras 2012 [74]	Predicting general health/disease burden (same as original)	Added depression to 2009 adaptation conditions and translated into Canadian French	Compared with CIRS two weeks after initial assessment, Pearson correlation coefficient=0.56 (0.38 to 0.70, *P*<0.01)
**Pope 2004 [25] The Centers for Medicare and Medicaid Services' Hierarchical Condition Category (CMS-HCC)**	Regular updates [75]	Predicting healthcare expenditure (same as original)	Updated annually with amended ICD-10 mappings and software	Regular reports; 2018 report lists detailed predictive expenditure accuracy for combinations of conditions of CMS-HCC.[69] Ratio of one-year predicted to actual expenditure=1.00 (but gives caveat that this is an average in a very large group)

Publication and name of index	Updates or adaptations	Aim compared to original index	Details	Performance or comparison
**Sangha 2003 [26] Self-Administered Comorbidity Questionnaire**	Hudson 2008 [76]	Predicting health-related quality of life (included in original)	Adapted for people with systemic autoimmune diseases: removed rheumatoid arthritis from condition list	Kendall's Tau b correlation between SCQ and a) PCS: Systemic sclerosis= -0.26, systemic lupus= -0.31 b) MCS: Systemic sclerosis= -0.14, systemic lupus= -0.12
	Sridharan 2014 [77]	Predicting mortality (different from original)	Adapted for patients with end-stage renal disease: added eight prevalent conditions in this group and space for free-text answers; removed question on kidney disease	AUC for 18-month mortality: modified SCQ=0.72 (0.65 to 0.80), Charlson=0.75 (0.68 to 0.82)
**Desai 2002 [28] High-Risk Diagnoses for the Elderly Scale (HRDES)**	Burden of Illness Score for Elderly Persons (BISEP) [78]	Predicting mortality (same as original)	Added serum albumin and creatinine, dementia and walking impairment to ten weighted conditions from HRDES	C-statistics for one year mortality in validation cohort: HRDES=0.59, BISEP=0.77
**Greenfield 1995 [31] Total Illness Burden Index (TIBI)**	TIBI-P [79]	Predicting health-related quality of life (same as original) and mortality (different)	Modified for men with prostate cancer. Reduced domains on some items, included diabetes and non-prostate cancers	R^2 for PCS: adjusted TIBI-P=0.35, demographics alone=0.16. 3.5-year mortality: adjusted HR for highest scoring group compared to lowest=13.1 (6.3 to 27.4) [80]
**Parkerson 1993 [32] Duke Severity of Illness Checklist (DUSOI)**	Duke Case-Mix System (DUMIX) [81]	Predicting healthcare expenditure (different from original)	Adds demographic information and self-reported functional health status to DUSOI	Variance in future clinic charges explained by DUMIX=17.1%, age + gender alone=9.1%
**Von Korff 1992 [33] Chronic Disease Score**	Rx-Risk [82]	Predicting healthcare expenditure (healthcare use included in original)	Weighted drug groups increased to 57 from 25 in CDS. Includes children. Widely used and validated	In original paper, cost variance explained (R^2): RxRisk=8.7%, CMS-HCCs=15.4%, ACGs=10.2%.<br>In later validation, C-statistics for one-year mortality: weighted RxRisk=0.79 (0.78 to 0.79) [83]
	Rx-Risk-V [84]	Predicting healthcare expenditure (healthcare use included in original)	Adapted for use in veteran population and updated to include newer drugs. Includes 45 drug classes	Original paper quotes variance explained (R^2) for concurrent costs=0.18 and prospective costs=0.10. Compared to other scales in original Farley paper (see eTable 9) [21]
	McGregor 2006 [85]	Predicting specific diagnoses (different from original)	Adaptations for nosocomial infectious diseases: MRSA and VRE. Combined index (CDS-ID) contains six conditions	C-statistics for predicting MRSA diagnosis: CDS-ID=0.57, CDS=0.52; for predicting VRE diagnosis: CDS-ID=0.64, CDS=0.57
**Starfield 1991 [34] Ambulatory Care Groups**	Regular ongoing updates [86]	Predicting healthcare expenditure (same as original)	Updates to disease markers, visit classifications and software. Version 12.0 released March 2019	See eTable 10
**Charlson 1987 [35]**	Deyo 1992 [87]	Predicting mortality (same as original) and other outcomes	Aligned conditions with ICD-9 codes. Combined leukaemia/lymphoma with other malignancies giving 17 conditions	Used as comparator in other studies – see eTables 9 and 10

Publication and name of index	Updates or adaptations	Aim compared to original index	Details	Performance or comparison
	Romano 1993 [88] (Dartmouth-Manitoba)	Predicting mortality (same as original)	Adapted for use with administrative data using ICD-9-CM codes; broader definitions than Deyo	Compared to other scales in original Farley paper (see eTable 9) [21]
	D'Hoore 1993 [89]	Predicting mortality (same as original)	Uses first three digits of ICD-9	In this paper, C-statistic for in-hospital mortality=0.83
	Ghali 1996 [90]	Predicting mortality (same as original)	Assigns new weights to Deyo's system, according to study-specific mortality. Includes only five conditions	In this paper, C-statistic for in-hospital mortality=0.74, original Charlson index=0.70
	Quan 2005 [91]	Predicting mortality (same as original)	Adapted for ICD-10, includes 12 conditions. Later revision assigns new weights to conditions [92]	In this paper, C-statistics discriminating in-hospital mortality in one cohort: Quan=0.83, original Charlson=0.81
**Linn 1968 [36]**<br>**Cumulative Illness Rating Scale (CIRS)**	CIRS-G [93]	Predicting overall illness severity (same as original) and functional impairment (different)	Modified for older population. Added haematopoietic category, clarified where to list dementia and breast disorders. Online calculators available with examples [94]	Widely used. This paper reports Spearman correlation between CIRS-G and a) OARS ADL=0.58 (P<0.02) and b) increasing overall medical impairment=0.45 (*P*=0.002)
	CIRS-SA [95]	Predicting overall illness severity (same as original)	Substance abuse version. Has 13 items including HIV status and guidance on where to record hepatitis. Removed psychiatry category	Cronbach's coefficient for internal consistency=0.57. Kendall's tau for agreement between overall CIRS-SA and consultant assessment of illness severity=0.58 (*P*<0.01)
	Mistry 2004 [96]	Predicting mortality (different from original)	Two subscales, including (CIRS-IP) and excluding (CIRS-PH) acute conditions	Cox proportional hazards regression for age-adjusted days of survival, standardised β CIRS-IP=0.55 (0.14-0.96), CIRS-PH=0.70 (0.28-1.11)

Abbreviations in eTable 11:
AUC: Area under the curve
BMI: Body mass index
EQ-5D: EuroQol five-dimension measure of health status
ICD-9-CM: International Classification of Diseases, Ninth Revision, Clinical Modification
ICD-10: International Statistical Classification of Diseases and Related Health Problems 10th Revision
MCS: Mental Component Score of SF-36
MRSA: Methicillin-resistant *Staphylococcus aureus*
OARS ADL: Older Americans Resources and Services Activities of Daily Living Scale
PCS: Physical Component Score of SF-36
SF-6D: Shortened revised version of SF-36
VRE: Vancomycin-resistant enterococci

Appendix lay summary - "Ways of measuring multiple conditions"

Title of research article: *Measuring multimorbidity beyond counting diseases: systematic review of community and population studies and guide to index choice.*

This research is funded by the Medical Research Foundation and the Medical Research Council through a grant to Dr Lucy Stirland.

What is a systematic review?

A systematic review is a common type of research. It's a way of finding lots of published research articles and summarising them together.

What do we know about this topic already?

It's common for people to have two or more chronic conditions at once. This is often called multimorbidity. Researchers and clinicians measure multimorbidity in many different ways.

What questions does this review ask?

1. What methods exist for measuring multimorbidity?
2. How good are they?
3. Do they include mental health?

How was the search carried out?

We decided in advance which topics to include. In October 2018, we searched seven online medical research databases. Two researchers separately checked 5,560 article titles. We discarded irrelevant articles.

What did the review find?

We ended up with 35 papers, each describing a way to measure multimorbidity. Most of them combined the number of chronic conditions with other things like age. Some counted people's prescribed drugs and others included medical test results.

Most of the tools aimed to predict health in some way. For example, 18 of them looked at death rates, 13 at hospitalisations and six at quality of life.

Nearly all the papers considered mental health, with 18 counting it as part of multimorbidity. Eleven measures aimed to predict some aspect of mental health.

Only one paper mentioned including patients in their research design.

How good were the papers?

We graded each paper according to set standards. Six were high quality, 22 were satisfactory and seven were low quality. Three of the papers didn't mention who funded their research. Four were funded by drug companies – this might make them biased. The other 28 papers had no funding bias.

What does this mean for patients?

These tools might help make other research more relevant to people with multiple conditions. For example, drug trials use very healthy people who are not like most patients. Researchers could use these tools to account for multimorbidity in a wider range of people.

Healthcare officials can also use the tools to predict how services will be used and plan how to fund them.

Appendix references

1 Scottish Intercollegiate Guidelines Network (SIGN). Methodology Checklist 3: Cohort Studies, Version 3.0. 2012.http://www.sign.ac.uk/checklists-and-notes.html

2 Corrao G, Rea F, Di Martino M, *et al.* Developing and validating a novel multisource comorbidity score from administrative data: a large population-based cohort study from Italy. *BMJ Open* 2017;**7**:e019503. doi:10.1136/bmjopen-2017-019503

3 Wen YC, Chen LK, Hsiao FY. Predicting mortality and hospitalization of older adults by the multimorbidity frailty index. *PLoS One* 2017;**12**:1–10. doi:10.1371/journal.pone.0187825

4 Stanley J, Sarfati D. The new measuring multimorbidity index predicted mortality better than Charlson and Elixhauser indices among the general population. *J Clin Epidemiol* 2017;**92**:99–110. doi:10.1016/J.JCLINEPI.2017.08.005

5 Wei MY, Kawachi I, Okereke OI, *et al.* Diverse Cumulative Impact of Chronic Diseases on Physical Health-Related Quality of Life: Implications for a Measure of Multimorbidity. *Am J Epidemiol* 2016;**184**:357–65. doi:10.1093/aje/kwv456

6 Robusto F, Lepore V, D'Ettorre A, *et al.* The Drug Derived Complexity Index (DDCI) Predicts Mortality, Unplanned Hospitalization and Hospital Readmissions at the Population Level. *PLoS One* 2016;**11**:e0149203. doi:10.1371/journal.pone.0149203

7 Lorem GF, Schirmer H, Emaus N. Health impact index. Development and validation of a method for classifying comorbid disease measured against self-reported health. *PLoS One* 2016;**11**:e0148830. doi:10.1371/journal.pone.0148830

8 Pati S, Hussain MA, Swain S, *et al.* Development and Validation of a Questionnaire to Assess Multimorbidity in Primary Care: An Indian Experience. *Biomed Res Int* 2016;:1–9. doi:10.1155/2016/6582487

9 Hong C, Atlas S, Ashburner J, *et al.* Evaluating a Model to Predict Primary Care Physician-Defined Complexity in a Large Academic Primary Care Practice-Based Research Network. *JGIM J Gen Intern Med* 2015;**30**:1741–7. doi:10.1007/s11606-015-3357-8

10 Wister A V, Levasseur M, Griffith LE, *et al.* Estimating multiple morbidity disease burden among older persons: a convergent construct validity study to discriminate among six chronic illness measures, CCHS 2008/09. *BMC Geriatr* 2015;**15**:12. doi:10.1186/s12877-015-0001-8

11 Brettschneider C, Leicht H, Bickel H, *et al.* Relative impact of multimorbid chronic conditions on health-related quality of life - results from the MultiCare Cohort Study. *PLoS One* 2013;**8**:e66742. doi:10.1371/journal.pone.0066742

12 Min L, Wenger N, Walling AM, *et al.* When Comorbidity, Aging, and Complexity of Primary Care Meet: Development and Validation of the Geriatric CompleXity of Care Index. *J Am Geriatr Soc* 2013;**61**:542–50. doi:10.1111/jgs.12160

13 Carey IM, Shah SM, Harris T, *et al.* A new simple primary care morbidity score predicted mortality and better explains between practice variations than the Charlson index. *J Clin Epidemiol* 2013;**66**:436–44. doi:10.1016/j.jclinepi.2012.10.012

14 Dong YH, Chang CH, Shau WY, *et al.* Development and validation of a pharmacy-based comorbidity measure in a population-based automated health care database. *Pharmacotherapy* 2013;**33**:126–36. doi:10.1002/phar.1176

15 Bernabeu-Wittel M, Ollero-Baturone M, Moreno-Gaviño L, *et al.* Development of a new predictive model for polypathological patients. The PROFUND index. *Eur J Intern Med* 2011;**22**:311–7. doi:10.1016/j.ejim.2010.11.012

16 Mukherjee B, Ou H-T, Wang F, *et al.* A new comorbidity index: The health-related quality

of life comorbidity index. *J Clin Epidemiol* 2011;**64**:309–19. doi:10.1016/j.jclinepi.2010.01.025

17 Newman AB, Boudreau RM, Naydeck BL, *et al.* A physiologic index of comorbidity: relationship to mortality and disability. *Journals Gerontol Ser A* 2008;**63**:603–9. doi:10.1093/gerona/63.6.603

18 Tooth L, Hockey R, Byles J, *et al.* Weighted multimorbidity indexes predicted mortality, health service use, and health-related quality of life in older women. *J Clin Epidemiol* 2008;**61**:151–9. doi:10.1016/j.jclinepi.2007.05.015

19 George J, Vuong T, Bailey MJ, *et al.* Development and validation of the medication-based disease burden index. *Ann Pharmacother* 2006;**40**:645–50. doi:10.1345/aph.1G204

20 Lee SJ, Lindquist K, Segal MR, *et al.* Development and Validation of a Prognostic Index for 4-Year Mortality in Older Adults. *JAMA J Am Med Assoc* 2006;**295**:801. doi:10.1001/jama.295.7.801

21 Farley JF, Harley CR, Devine JW. A Comparison of Comorbidity Measurements to Predict Healthcare Expenditures. *Am J Manag Care* 2006;**12**:110–7.

22 Byles JE, D'Este C, Parkinson L, *et al.* Single index of multimorbidity did not predict multiple outcomes. *J Clin Epidemiol* 2005;**58**:997–1005. doi:10.1016/j.jclinepi.2005.02.025

23 Bayliss EA, Ellis JL, Steiner JF. Subjective assessments of comorbidity correlate with quality of life health outcomes: Initial validation of a comorbidity assessment instrument. *Health Qual Life Outcomes* 2005;**3**:51. doi:10.1186/1477-7525-3-51

24 Selim AJ, Fincke G, Ren XS, *et al.* Comorbidity assessments based on patient report: results from the Veterans Health Study. *J Ambul Care Manage* 2004;**27**:281–95. doi:10.1097/00004479-200407000-00011

25 Pope GC, Kautter J, Ellis RP, *et al.* Risk adjustment of Medicare capitation payments using the CMS-HCC model. *Health Care Financ Rev* 2004;**25**:119–41. doi:hcfr-25-4-119 [pii]

26 Sangha O, Stucki G, Liang MH, *et al.* The Self-Administered Comorbidity Questionnaire: A new method to assess comorbidity for clinical and health services research. *Arthritis Rheum* 2003;**49**:156–63. doi:10.1002/art.10993

27 Fan VS, Au D, Heagerty P, *et al.* Validation of case-mix measures derived from self-reports of diagnoses and health. *J Clin Epidemiol* 2002;**55**:371–80. doi:10.1016/S0895-4356(01)00493-0

28 Desai MM, Bogardus ST, Williams CS, *et al.* Development and Validation of a Risk-Adjustment Index for Older Patients: The High-Risk Diagnoses for the Elderly Scale. *J Am Geriatr Soc* 2002;**50**:474–81. doi:10.1046/j.1532-5415.2002.50113.x

29 Crabtree HL, Gray CS, Hildreth AJ, *et al.* The Comorbidity Symptom Scale: A Combined Disease Inventory and Assessment of Symptom Severity. *J Am Geriatr Soc* 2000;**48**:1674–8. doi:10.1111/j.1532-5415.2000.tb03882.x

30 Hornbrook MC, Goodman MJ. Chronic disease, functional health status, and demographics: a multi-dimensional approach to risk adjustment. *Health Serv Res* 1996;**31**:283–307.

31 Greenfield S, Sullivan L, Dukes KA, *et al.* Development and testing of a new measure of case mix for use in office practice. *Med Care* 1995;**33**:AS47–55.

32 Parkerson Jr GR, Broadhead WE, Tse C-KKJ. The Duke severity of illness checklist (DUSOI) for measurement of severity and comorbidity. *J Clin Epidemiol* 1993;**46**:379–93. doi:10.1016/0895-4356(93)90153-R

33 Von Korff M, Wagner EH, Saunders K. A Chronic Disease Score from automated pharmacy data. *J Clin Epidemiol* 1992;**45**:197–203. doi:10.1016/0895-4356(92)90016-G

34 Starfield B, Weiner J, Mumford L, *et al.* Ambulatory care groups: a categorization of diagnoses for research and management. *Health Serv Res* 1991;**26**:53–74.

35 Charlson ME, Pompei P, Ales KL, *et al.* A New Method of Classifying Prognostic Comorbidity in Longitudinal Studies: Development and Validation. *J Chronic Dis* 1987;**40**:373–83. doi:10.1016/0021-9681(87)90171-8

36 Linn BS, Linn MW, Gurel L. Cumulative Illness Rating Scale. *J Am Geriatr Soc* 1968;**16**:622–6. doi:10.1111/j.1532-5415.1968.tb02103.x

37 Fincke G, Spiro AI, Selim A. Measuring disease severity in the Veterans Health Study. In: Kazis L, Miller D, Skinner K, *et al.*, eds. *The health-related quality of life in veterans*. Washington, DC: : Department of Veterans Affairs 1995.

38 Wei MY, Kabeto MU, Langa KM, *et al.* Multimorbidity and physical and cognitive function: Performance of a new Multimorbidity-Weighted Index. *Journals Gerontol Ser A* 2018;**73**:225–32. doi:10.1093/gerona/glx114

39 Cortaredona S, Pambrun E, Verdoux H, *et al.* Comparison of pharmacy-based and diagnosis-based comorbidity measures from medical administrative data. *Pharmacoepidemiol Drug Saf* 2017;**26**:402–11. doi:10.1002/pds.4146

40 Bernabeu-Wittel M, Moreno-Gaviño L, Ollero-Baturone M, *et al.* Validation of PROFUND prognostic index over a four-year follow-up period. *Eur J Intern Med* 2016;**36**:20–4. doi:10.1016/J.EJIM.2016.07.022

41 Díez-Manglano J, del Corral Beamonte E, Ramos Ibáñez R, *et al.* Reliability of the PROFUND index to predict 4-year mortality in polypathological patients. *Med Clínica (English Ed* 2016;**147**:238–44. doi:10.1016/J.MEDCLE.2016.10.016

42 Díez-Manglano J, Cabrerizo García JL, García-Arilla Calvo E, *et al.* External validation of the PROFUND index in polypathological patients from internal medicine and acute geriatrics departments in Aragón. *Intern Emerg Med* 2015;**10**:915–26. doi:10.1007/s11739-015-1252-2

43 López-Garrido MA, Antequera Martín-Portugués I, Becerra-Muñoz VM, *et al.* Prevalence of comorbidities and the prognostic value of the PROFUND index in a hospital cardiology unit. *Rev Clínica Española (English Ed* 2017;**217**:87–94. doi:10.1016/J.RCENG.2016.10.006

44 Mehta HB, Sura SD, Sharma M, *et al.* Comparative performance of diagnosis-based and prescription-based comorbidity scores to predict health-related quality of life. *Med Care* 2016;**54**:519–27. doi:10.1097/MLR.0000000000000517

45 Ou H-T, Mukherjee B, Erickson SR, *et al.* Comparative Performance of Comorbidity Indices in Discriminating Health-related Behaviors and Outcomes. *Health Outcomes Res Med* 2011;**2**:e91–104. doi:10.1016/J.EHRM.2011.06.002

46 Beloosesky Y, Weiss A, Mansur N. Validity of the Medication-Based Disease Burden Index Compared with the Charlson Comorbidity Index and the Cumulative Illness Rating Scale for Geriatrics. *Drugs Aging* 2011;**28**:1007–14. doi:10.2165/11597040-000000000-00000

47 Vitry A, Wong SA, Roughead EE, *et al.* Validity of medication-based co-morbidity indices in the Australian elderly population. *Aust N Z J Public Health* 2009;**33**:126–30. doi:10.1111/j.1753-6405.2009.00357.x

48 Cruz M, Covinsky K, Widera EW, *et al.* Predicting 10-Year Mortality for Older Adults. *JAMA* 2013;**309**:874. doi:10.1001/jama.2013.1184

49 Froehner M, Koch R, Novotny V, *et al.* Lee mortality index as comorbidity measure in patients undergoing radical cystectomy. *Springerplus* 2015;**4**:55. doi:10.1186/s40064-015-0834-9

50 Tyack Z, Kuys S, Cornwell P, *et al.* Reproducibility, longitudinal validity and interpretability of the Disease Burden Morbidity Assessment in people with chronic disease. *Chronic Illn* 2018;**14**:310–25. doi:10.1177/1742395318789469

51 Wijers IG, Ayala A, Rodriguez-Blazquez C, *et al.* The Disease Burden Morbidity Assessment in older adults and its association with mortality and other health outcomes. *Eur J Ageing* 2018;:1–11. doi:10.1007/s10433-018-0491-2

52 Wijers IGM, Ayala A, Rodriguez-Blazquez C, *et al.* Rasch Analysis and Construct Validity of the Disease Burden Morbidity Assessment in Older Adults. *Gerontologist* 2018;**58**:e302–10. doi:10.1093/geront/gnx061

53 Li P, Kim MM, Doshi JA. Comparison of the performance of the CMS Hierarchical Condition Category (CMS-HCC) risk adjuster with the Charlson and Elixhauser comorbidity measures in predicting mortality. *BMC Health Serv Res* 2010;**10**:245. doi:10.1186/1472-6963-10-245

54 Haas LR, Takahashi PY, Shah ND, *et al.* Risk-stratification methods for identifying patients for care coordination. *Am J Manag Care* 2013;**19**:725–32.

55 Pope GC, Kautter J, Ingber MJ, *et al.* Evaluation of the CMS-HCC Risk Adjustment Model. Baltimore, Maryland, USA: 2011.

56 Olomu AB, Corser WD, Stommel M, *et al.* Do self-report and medical record comorbidity data predict longitudinal functional capacity and quality of life health outcomes similarly? *BMC Health Serv Res* 2012;**12**:398. doi:10.1186/1472-6963-12-398

57 Stolwijk C, van Tubergen A, Ramiro S, *et al.* Aspects of validity of the self-administered comorbidity questionnaire in patients with ankylosing spondylitis. *Rheumatology* 2014;**53**:1054–64. doi:10.1093/rheumatology/ket354

58 Parkerson Jr GR, Harrell Jr FE, Hammond WE, *et al.* Characteristics of Adult Primary Care Patients as Predictors of Future Health Services Charges. *Med Care* 2001;**39**:1170–81.

59 Shiels C, Eccles M, Hutchinson A, *et al.* The inter-rater reliability of a generic measure of severity of illness. *Fam Pract* 1997;**14**:466–71. doi:10.1093/fampra/14.6.466

60 Clark DO, Von Korff M, Saunders K, *et al.* A chronic disease score with empirically derived weights. Med. Care. 1995;**33**:783–95. doi:10.1097/00005650-199508000-00004

61 Starfield B, Kinder K. Multimorbidity and its measurement. *Health Policy (New York)* 2011;**103**:3–8. doi:10.1016/J.HEALTHPOL.2011.09.004

62 Weir S, Aweh G, Clark RE. Case selection for a Medicaid chronic care management program. *Health Care Financ Rev* 2008;**30**:61–74.

63 Austin PC, van Walraven C, Wodchis WP, *et al.* Using the Johns Hopkins Aggregated Diagnosis Groups (ADGs) to predict mortality in a general adult population cohort in Ontario, Canada. *Med Care* 2011;**49**:932–9. doi:10.1097/MLR.0b013e318215d5e2

64 Yurkovich M, Avina-Zubieta JA, Thomas J, *et al.* A systematic review identifies valid comorbidity indices derived from administrative health data. *J Clin Epidemiol* 2015;**68**:3–14. doi:10.1016/J.JCLINEPI.2014.09.010

65 Waldman E, Potter JF. A prospective evaluation of the cumulative illness rating scale. *Aging Clin Exp Res* 1992;**4**:171–8. doi:10.1007/BF03324087

66 Parmelee PA, Thuras PD, Katz IR, *et al.* Validation of the Cumulative Illness Rating Scale in a Geriatric Residential Population. *J Am Geriatr Soc* 1995;**43**:130–7. doi:10.1111/j.1532-5415.1995.tb06377.x

67 Nagaratnam N, Gayagay G. Validation of the Cumulative Illness Rating Scale (CIRS) in hospitalized nonagenarians. *Arch Gerontol Geriatr* 2007;**44**:29–36. doi:10.1016/J.ARCHGER.2006.02.002

68 Fortin M, Hudon C, Dubois M-F, *et al.* Comparative assessment of three different indices of multimorbidity for studies on health-related quality of life. *Health Qual Life Outcomes* 2005;**3**:74. doi:10.1186/1477-7525-3-74

69 Ou H-T, Lin C-Y, Erickson SR, *et al.* Refined comorbidity index based on dimensionality of comorbidity for use in studies of health-related quality of life. *Qual Life Res* 2016;**25**:2543–57. doi:10.1007/s11136-016-1306-6

70 Sanders JL, Boudreau RM, Penninx BW, *et al.* Association of a Modified Physiologic Index With Mortality and Incident Disability: The Health, Aging, and Body Composition Study. *Journals Gerontol Ser A* 2012;**67**:1439–46. doi:10.1093/gerona/gls123

71 Sanders JL, Minster RL, Barmada MM, *et al.* Heritability of and Mortality Prediction With a Longevity Phenotype: The Healthy Aging Index. *Journals Gerontol Ser A Biol Sci Med Sci* 2014;**69**:479–85. doi:10.1093/gerona/glt117

72 Kobayashi LC, Jackson SE, Lee SJ, *et al.* The development and validation of an index to predict 10-year mortality risk in a longitudinal cohort of older English adults. *Age Ageing* 2016;**46**:427–32. doi:10.1093/ageing/afw199

73 Bayliss EA, Ellis JL, Steiner JF. Seniors' self-reported multimorbidity captured biopsychosocial factors not incorporated into two other data-based morbidity measures. *J Clin Epidemiol* 2009;**62**:550-557.e1. doi:10.1016/J.JCLINEPI.2008.05.002

74 Poitras M-E, Fortin M, Hudon C, *et al.* Validation of the disease burden morbidity assessment by self-report in a French-speaking population. *BMC Health Serv Res* 2012;**12**:35. doi:10.1186/1472-6963-12-35

75 U.S. Centers for Medicare & Medicaid Services. Risk Adjustment. CMS.gov. 2018.https://www.cms.gov/Medicare/Health-Plans/MedicareAdvtgSpecRateStats/Risk-Adjustors.html (accessed 28 Mar 2019).

76 Hudson M, Bernatsky S, Taillefer S, *et al.* Patients with systemic autoimmune diseases could not distinguish comorbidities from their index disease. *J Clin Epidemiol* 2008;**61**:654–62. doi:10.1016/J.JCLINEPI.2007.08.010

77 Sridharan S, Berdeprado J, Vilar E, *et al.* A self-report comorbidity questionnaire for haemodialysis patients. *BMC Nephrol* 2014;**15**:134. doi:10.1186/1471-2369-15-134

78 Inouye SK, Bogardus ST, Vitagliano G, *et al.* Burden of Illness Score for Elderly Persons: Risk adjustment incorporating the cumulative impact of diseases, physiologic abnormalities, and functional impairments. *Med Care* 2003;**41**:70–83. doi:10.1097%2F00005650-200301000-00010

79 Stier DM, Greenfield S, Lubeck DP, *et al.* Quantifying comorbidity in a disease-specific cohort: adaptation of the total illness burden index to prostate cancer. *Urology* 1999;**54**:424–9. doi:10.1016/S0090-4295(99)00203-4

80 Litwin MS, Greenfield S, Elkin EP, *et al.* Assessment of prognosis with the total illness burden index for prostate cancer. *Cancer* 2007;**109**:1777–83. doi:10.1002/cncr.22615

81 Parkerson Jr GR, Michener JL, Yarnall KS., *et al.* Duke Case-Mix System (DUMIX) for Ambulatory Health Care. *J Clin Epidemiol* 1997;**50**:1385–94. doi:10.1016/S0895-4356(97)00217-5

82 Fishman PA, Goodman MJ, Hornbrook MC, *et al.* Risk adjustment using automated ambulatory pharmacy data the RxRisk model. *Med Care* 2003;**41**:84–99. doi:10.1097/00005650-200301000-00011

83 Pratt NL, Kerr M, Barratt JD, *et al.* The validity of the Rx-Risk Comorbidity Index using medicines mapped to the Anatomical Therapeutic Chemical (ATC) Classification System. *BMJ Open* 2018;**8**:e021122. doi:10.1136/bmjopen-2017-021122

84 Sloan KL, Sales AE, Liu C-F, *et al.* Construction and Characteristics of the RxRisk-V: A

VA-Adapted Pharmacy-Based Case-Mix Instrument. *Med Care* 2003;**41**:761–74.

85 McGregor JC, Perencevich EN, Furuno JP, *et al.* Comorbidity risk-adjustment measures were developed and validated for studies of antibiotic-resistant infections. *J Clin Epidemiol* 2006;**59**:1266–73. doi:10.1016/J.JCLINEPI.2006.01.016

86 The Johns Hopkins University. The Johns Hopkins ACG® System. 2019.https://www.hopkinsacg.org/ (accessed 28 Mar 2019).

87 Deyo RA, Cherkin DC, Ciol MA. Adapting a clinical comorbidity index for use with ICD-9-CM administrative databases. *J Clin Epidemiol* 1992;**45**:613–9. doi:10.1016/0895-4356(92)90133-8

88 Romano PS, Roos LL, Jollis JG. Adapting a clinical comorbidity index for use with ICD-9-CM administrative data: differing perspectives. *J Clin Epidemiol* 1993;**46**:1075–9. doi:10.1016/0895-4356(93)90103-8

89 D'Hoore W, Sicotte C, Tilquin C. Risk adjustment in outcome assessment: the Charlson comorbidity index. *Methods Inf Med* 1993;**32**:382–7.

90 Ghali WA, Hall RE, Rosen AK, *et al.* Searching for an improved clinical comorbidity index for use with ICD-9-CM administrative data. *J Clin Epidemiol* 1996;**49**:273–8. doi:10.1016/0895-4356(95)00564-1

91 Quan H, Sundararajan V, Halfon P, *et al.* Coding Algorithms for Defining Comorbidities in ICD-9-CM and ICD-10 Administrative Data. *Med Care* 2005;**43**:1130–9. doi:10.1097/01.mlr.0000182534.19832.83

92 Quan H, Li B, Couris CM, *et al.* Updating and Validating the Charlson Comorbidity Index and Score for Risk Adjustment in Hospital Discharge Abstracts Using Data From 6 Countries. *Am J Epidemiol* 2011;**173**:676–82. doi:10.1093/aje/kwq433

93 Miller MD, Paradis CF, Houck PR, *et al.* Rating chronic medical illness burden in geropsychiatric practice and research: Application of the Cumulative Illness Rating Scale. *Psychiatry Res* 1992;**41**:237–48. doi:10.1016/0165-1781(92)90005-N

94 MDCalc. Cumulative Illness Rating Scale-Geriatric (CIRS-G). 2019.https://www.mdcalc.com/cumulative-illness-rating-scale-geriatric-cirs-g

95 Castillo C, Bulbena A, Serras E, *et al.* Medical Assessment in Drug Addicts: Reliability and Validity of the Cumulative Illness Rating Scale (Substance Abuse Version). *Eur Addict Res* 2004;**10**:112–7. doi:10.1159/000077699

96 Mistry R, Gokhman I, Bastani R, *et al.* Measuring Medical Burden Using CIRS in Older Veterans Enrolled in UPBEAT, a Psychogeriatric Treatment Program: A Pilot Study. *Journals Gerontol Ser A Biol Sci Med Sci* 2004;**59**:M1068–75. doi:10.1093/gerona/59.10.M1068
